# Effects of laryngeal mask airway removal under different anesthesia states on pediatric airway complications: a systematic review and meta-analysis

**DOI:** 10.7717/peerj.21551

**Published:** 2026-07-31

**Authors:** Minhui Jiang, Zhihui Ma, Guanyu Chen, Shiyu Shu

**Affiliations:** 1 The Department of Anesthesiology, The Second Affiliated Hospital of Chongqing Medical University, ChongQing, China; 2 The Department of Anesthesiology, Zigong Fourth People’s Hospital, Zigong, China

**Keywords:** Children, Anesthesia, Laryngeal mask airway (LMA), Removal, Airway complication, Meta-analysis

## Abstract

**Objective:**

This meta-analysis was designed to compare airway complication rates between deep *vs*. awake laryngeal mask airway (LMA) removal in pediatric non-airway surgery, while discussing the impact of anesthetic agents, patient position and age.

**Methods:**

We systematically searched PubMed, Web of Science, and Cochrane Library to identify studies meeting the inclusion criteria. The primary outcome measured was the incidence of overall airway complications, which included laryngospasm, airway obstruction, desaturation, breath-holding, cough, and excessive secretions. Secondary outcomes included subgroup analyses stratified by patient positioning during LMA removal, inhalational anesthetic agents and age.

**Results:**

Pooled analysis of 10 randomized controlled trials (RCTs) (*n* = 1,713) revealed no significant difference in overall airway complications (relative risk (RR) of 0.93, 95% confidence interval (CI) [0.65–1.32], *p* = 0.68) or specific outcomes (laryngospasm, desaturation, breath-holding, cough) between deep and awake LMA removal; however, deep removal was associated with a significantly higher airway obstruction risk and a lower incidence of excessive secretions. Subgroup analyses demonstrated that inhalational anesthetic agents type (sevoflurane/isoflurane) did not alter most complications but universally increased airway obstruction risk, whereas positioning during deep removal reduced secretions in both lateral/supine positions yet increased obstruction, with lateral positioning uniquely lowering desaturation risk. Notably, age did not modulate the airway obstruction risk profile: children under 6 years and over 6 years exhibited comparably elevated obstruction risks with deep removal.

**Conclusions:**

This meta-analysis suggests awake LMA removal is associated with lower airway obstruction risk than deep removal, which primarily reduces secretions. Given the higher clinical severity of obstruction, clinicians may prioritize its prevention. This finding appears consistent across age groups, though caution is advised for infants (<2 years) due to limited data. Lateral positioning may mitigate desaturation during deep removal. We suggest risk-stratified protocols, with lateral positioning and monitoring advised if deep removal is performed.

## Introduction

The laryngeal mask airway (LMA) has become a standard tool for airway management in pediatric anesthesia, serving as an effective alternative to endotracheal tubes (ETTs) and face masks. Its use in pediatric procedures has increased significantly over the past decade due to easier insertion and fewer complications compared to ETTs (Brain, 1983; Joshi et al., 1997; Pennant & White, 1993; Liu et al., 2021; Yu & Beirne, 2010; Yang et al., 2024), with ample literature validating its safety and efficacy (Pennant & White, 1993; Liu et al., 2021; Yu & Beirne, 2010).

However, the optimal timing for LMA removal—under deep anesthesia or when the patient is awake—remains controversial, especially in non-airway surgeries (Mathew & Mathew, 2015). Deep removal is associated with airway obstruction and desaturation (Kitching, Walpole & Blogg, 1996; Samarkandi, 1998), while awake removal may trigger laryngospasm, breath-holding, coughing, or excess secretions (Pappas et al., 2001), exacerbated by incomplete sedation (Stevanovic et al., 2015).

Inhalational anesthetics like sevoflurane and isoflurane are commonly used. Sevoflurane is preferred for rapid induction/emergence and non-irritating airway effects (Epstein et al., 1995; Kangralkar & Jamale, 2021), supported by its favorable hemodynamic profile and reduced airway reactivity compared to isoflurane in children (Brioni et al., 2017). Pappas et al. (2001) found that with sevoflurane, anesthetic depth during LMA removal does not affect airway hyperreactivity, but with isoflurane, awake removal increases adverse events, highlighting the impact of agent choice. Age and patient position also matter. Younger children (<2 years) face higher laryngospasm risks due to smaller airways and immature reflexes, while older ones may have more coughing or agitation. Supine position increases obstruction risk, lateral position reduces aspiration, and semi-recumbent position may improve respiratory mechanics.

This study thus analyzes airway complications of deep *vs*. awake LMA removal, assessing the impacts of anesthetics (sevoflurane *vs*. isoflurane), position, and age.

## Methods

This study was performed according to the Preferred Reporting Items for Systematic Reviews and Meta-Analyses (PRISMA) statement. The review protocol was registered on the INPLASY website (registration number: INPLASY202220022 https://inplasy.com/inplasy-2022-2-0022/).

### Search for trials

A systematic literature search was conducted across PubMed, Web of Science, and the Cochrane Library, covering publications from database inception until June 7, 2026. Tailored search strategies incorporating the keywords “children,” “anesthesia,” “laryngeal mask airway (LMA),” and “removal” were applied to each database. Boolean operators structured the search syntax: ((children OR child OR kid OR kids OR pediatric) AND (anesthesia) AND (laryngeal mask OR LMA) AND (removal OR extubation)). Supplementary searches included dissertations, reference lists, and grey literature. Two independent reviewers executed the search and resolved disagreements through discussion.

### Inclusion and exclusion criteria

Studies were included if they met the following criteria: (1) randomized controlled trials (RCTs) examining airway complications after LMA removal; (2) pediatric patients undergoing non-airway surgeries; (3) comparison of deep *vs*. awake anesthesia removal. Exclusion criteria encompassed case reports, conference abstracts, review articles, studies using halothane anesthesia, and duplicate publications (retaining only the most recent version).

### Data collection and outcome

Two investigators independently extracted data using standardized forms. Collected data included: study characteristics (authors, publication year), patient demographics (age), American Society of Anesthesiologists (ASA) physical status, and detailed documentation of LMA removal-related airway complications stratified by anesthesia depth (deep *vs*. awake). Discrepancies were reconciled through discussion. Primary endpoint was the frequency of total respiratory complications. This composite outcome encompassed several clinical events: laryngospasm (defined as partial or total blockage of the airway at the laryngeal level), general airway obstruction, and oxygen desaturation, characterized by SpO_2_ levels falling below 90–95% while in the post-anesthesia care unit (PACU). Additionally, it included breath-holding, excessive secretions, and coughing—specifically identified as a vigorous, persistent cough lasting longer than 10 s post-anesthesia. For secondary outcomes, we conducted subgroup analyses to investigate the influence of patient positioning during LMA removal and the specific type of volatile anesthetic used, such as sevoflurane *vs*. isoflurane.

### Risk of bias assessment

To determine methodological quality, two authors independently applied the Cochrane Risk of Bias Tool across six criteria: random sequence generation, allocation concealment, blinding, incomplete outcome data, selective reporting, and other biases. The bias risk for each parameter was graded as “low,” “high,” or “unclear.” Any inter-rater disagreements were resolved *via* consensus discussion.

### Statistical analysis

Statistical computations were performed using STATA 14.0 (StataCorp, TX). We calculated relative ratios (RRs) and 95% confidence intervals (CIs) to evaluate outcomes. Heterogeneity was quantified *via* the I^2^ statistic, utilizing a random-effects model for I^2^ ≥ 50% and a fixed-effects model for values below this threshold. Subgroup analyses investigated age, patient positioning, and anesthetic agents. To ensure robustness, leave-one-out sensitivity analyses were executed. Potential publication bias was appraised through funnel plots and Begg’s/Egger’s tests. A *p*-value < 0.05 was considered statistically significant.

## Results

### Characteristics of trials and patients

**Figure 1:**
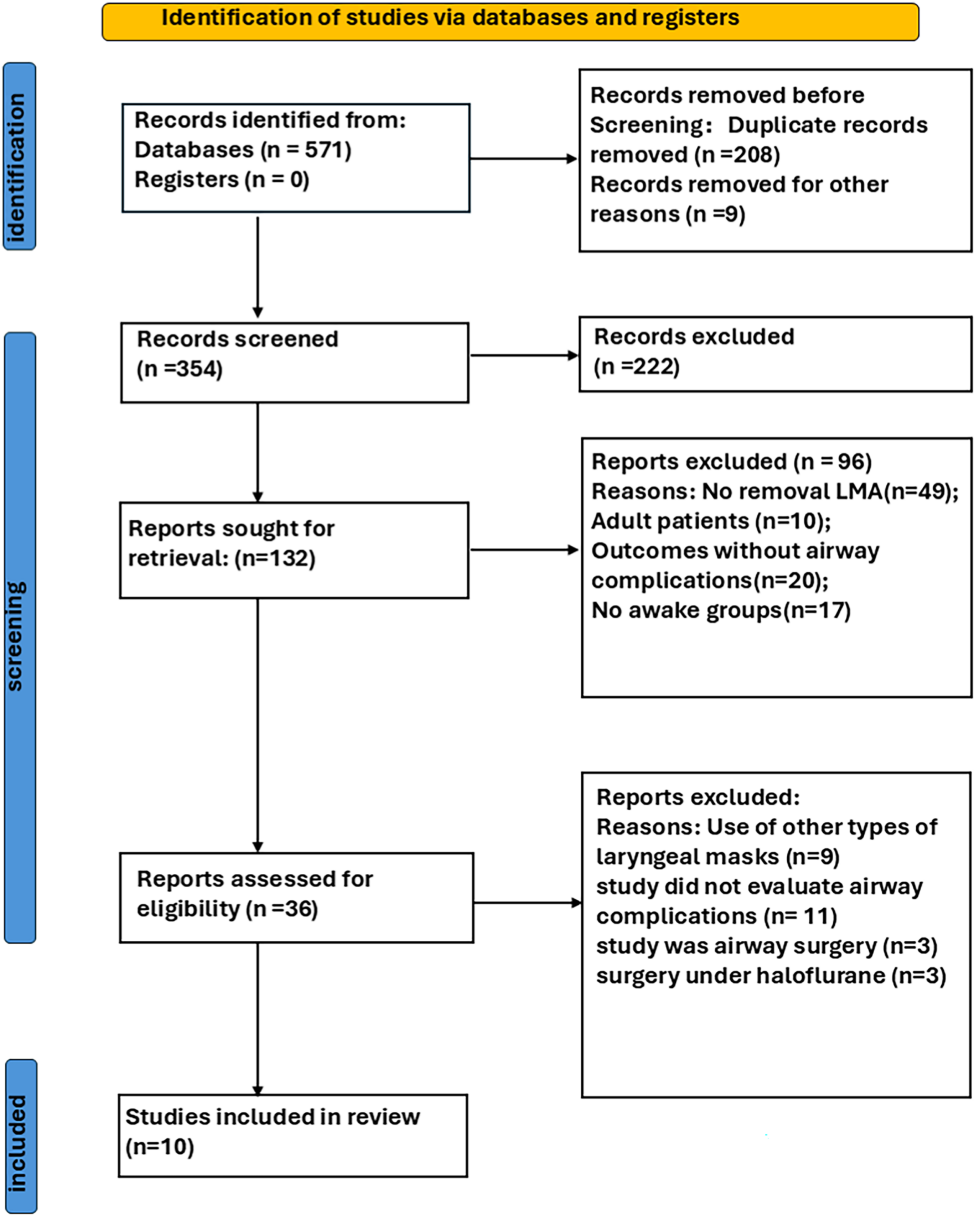
Preferred reporting items for systematic reviews and meta-analyses (PRISMA) flow diagram and summarizes the reasons for the exclusion of studies.

A total of 571 studies were initially identified from database searches. After removing 208 duplicates, 222 records were excluded through title/abstract screening, followed by exclusion of 26 additional studies during full-text review (Fig. 1).

Table 1 summarizes characteristics of the included RCTs (*n* = 10) (Park et al., 2012; Lerman et al., 2010; Abbasi, Siddiqui & Qamar-Ul-Hoda, 2022; Kumar et al., 2023; Sun et al., 2021; Thomas-Kattappurathu, Kasisomayajula & Short, 2015; Trachsel et al., 2022; Sinha & Sood, 2006; Baird, Mayor & Goodwin, 1999; Chhikara & Seelwal, 2024). Patients underwent diverse procedures: infraumbilical surgeries, herniotomy, ophthalmic and orthopedic operations. Sample sizes ranged from 85 to 382 across studies, with age distributions reported separately for deep and awake removal groups. Most participants were ASA physical status I–II (some included status I–III). Inhalational anesthetics comprised sevoflurane (*n* = 6), isoflurane (*n* = 3), or desflurane-isoflurane combinations (*n* = 1). Adjunctive agents included midazolam, N_2_O, fentanyl, and regional blocks. Patient positioning encompassed lateral (*n* = 4), supine (*n* = 5), and unspecified (*n* = 1). Primary endpoints focused on airway complications: cough, laryngospasm, oxygen desaturation (SpO_2_ <90–95%), breath-holding, and excessive secretions.

**Table 1:** Patient characteristics, anesthetics, and endpoints in the included RCT.

	Author	Year	Type of surgery	Number of patients (deep/awake)	Age (year)		ASA status	Inhalational anesthetic	Other anesthetics agent	Position	Endpoints
					Deep	Awake					
1	Pappas et al. (2001)	2001	Infraumbilical procedures	119 (59/60)	3.3	2.9	I–II	Sevoflurane and isoflurane	Midazolam, N2O	Lateral	Airway hyperreactivity, SpO2 <90%
2	Sinha & Sood (2006)	2006	Hemiotomy, orchidopexy or plastic surgery of lower limbs	125 (66/59)	2.6	2.7	I–II	Sevoflurane	N2O, fentanyl local block	Lateral	Desaturation (SpO2 <95%), and coughing, salivation, laryngospasm
3	Kumar et al. (2023)	2023	Paediatric surgery Plastic surgery Opthalmic surgery Orthopaedic surgery	140 (70/70)	8.2	7.4	I–II	Sevoflurane	Not reported	Lateral and supine	Desaturation <90%, laryngospasm, airway obstruction, High secretions
4	Thomas-Kattappurathu, Kasisomayajula & Short (2015)	2015	Daycase surgery: Paediatric surgery; Orthopaedic surgery; Ophthalmic surgery; Plastic surgery	212 (106/106)	7.7	6.8	I–II	Sevoflurane	Propofol supplementary analgesia (including opioids)	Lateral and supine	Desaturation <90%, laryngospasm, airway obstruction, excessive secretions
5	Park et al. (2012)	2012	Inguinal hernia repair or hydrocelectomy	85 (42/43)	4.2	3.9	I–II	Sevoflurane	Local block	/	Excessive secretion, cough, breath-holding, laryngospasm, SpO2 <95%, airway obstruction
6	Lerman et al. (2010)	2010	Ambulatory surgery	364 (176/188)	7.4	7.5	I–III	Desflurane and isoflurane	Midazolam, N2O, propofol	/	Airway obstruction, breath-holding, cough, laryngospasm excessive secretion, desaturation
7	Baird, Mayor & Goodwin (1999)	1999	Elective day-case surgery	90 (45/45)	7	7.8	Not reported	Isoflurane	Propofol, fentanyl, N20 paracetamol or local block	Lateral	Airway obstruction, cough, desaturation (SpO2 <96% SpO2 <94%, SpO2 <91%)
8	Chhikara & Seelwal (2024)	2024	Elective minor urogenital, pelvic, and lower limb surgery	90 (45/45)	4.51	5.16	I–II	Isoflurane	Glycopyrrolate, ondansetron, fentanyl, propofol, N20 caudal epidural block	Lateral	Excessive salivation, laryngospasm, cough, SpO2 <95%
9	Sun et al. (2021)	2017	Selective squint correction surgery	382 (191/191)	3.9	3.9	I–II	Sevoflurane	Propofol, sufentanil	Supine	Cough, laryngospasm SpO2 <96%
10	Abbasi, Siddiqui & Qamar-Ul-Hoda (2022)	2022	Elective infraumbilical surgeries	106 (53/53)	2.5	2.1	I–II	Isoflurane	Propofol, N2O local block	/	Laryngospasm, airway obstruction, cough, SpO2 <95%

### Methodological quality and risk of bias

The methodological quality of the included studies was evaluated using the Cochrane Risk of Bias Tool, with detailed assessments illustrated in Fig. S19. While patients were randomly assigned to groups, the randomization method was not clearly described in the study by Lerman. Three studies provided a clear description of the allocation concealment process. Most studies displayed unclear or high risk of performance bias, except for those conducted by Pappas and Sun. Seven studies reported complete outcome data, and active reporting was assessed as low risk in eight studies. The risk of other biases was rated low in five studies.

### Primary outcome

Pooled analysis of 1,713 pediatric patients across 10 RCTs revealed no significant difference in overall airway complications between deep and awake LMA removal (RR 0.93, 95% CI [0.65–1.32], *p* = 0.68), despite high heterogeneity (I^2^ = 80.1%) (Fig. S1).

**Figure 2:**
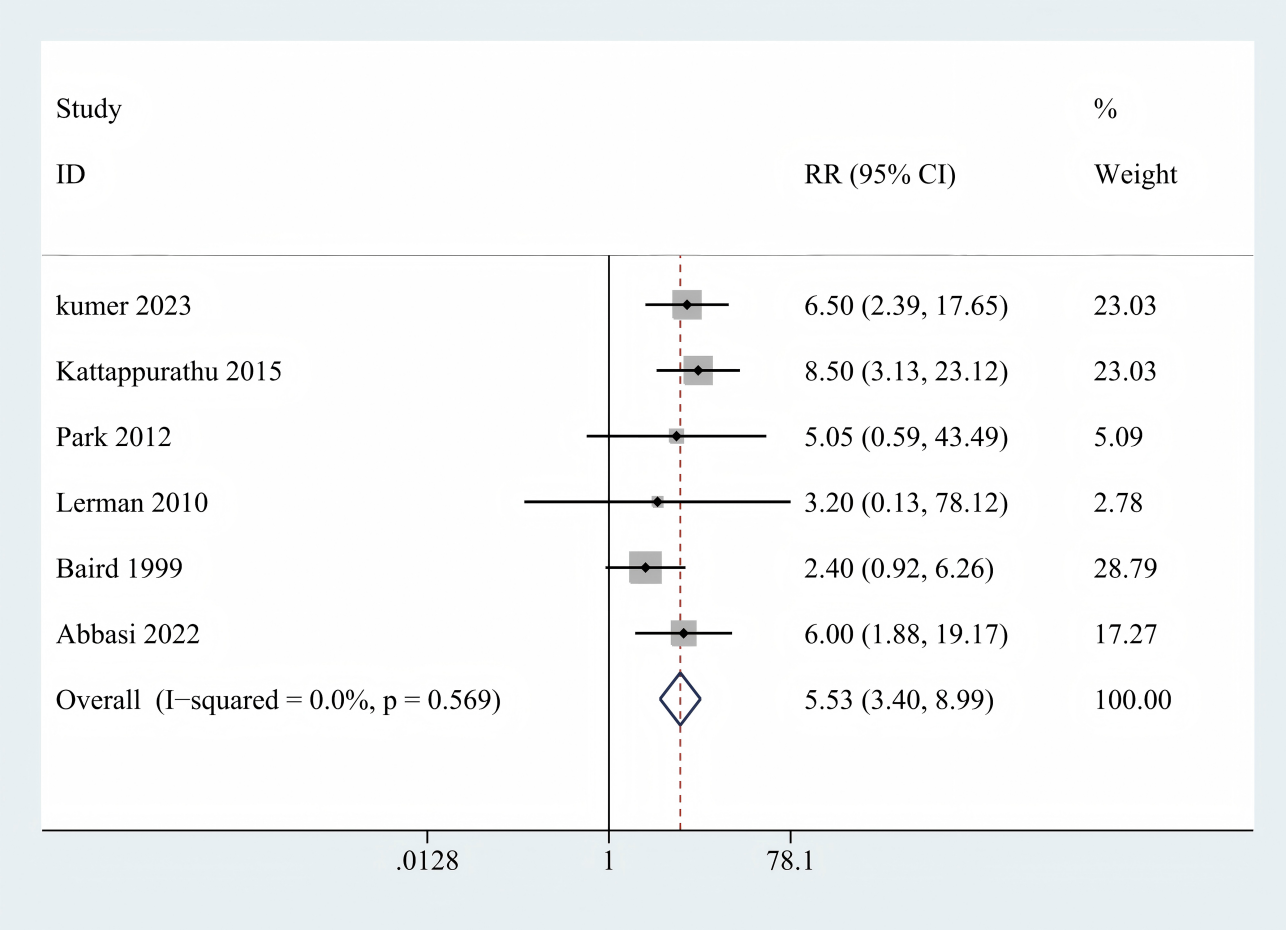
Forest plot of comparison between deep removal (experimental) and awake removal (control) for the outcome of airway obstruction.

Airway obstruction risk was significantly elevated with deep removal (RR 5.53, 95% CI [3.40–8.99], *p* < 0.001; I^2^ = 0%) (Fig. 2), while excessive secretions were substantially reduced (RR 0.04, 95% CI [0.01–0.13], *p* < 0.001; I^2^ = 0%) (Fig. S16). There was no difference in the incidence of other airway-related complications such as laryngospasm (RR 1.19, 95% CI [0.72–1.95], *p* = 0.49; I² = 31.5%) (Fig. S5), desaturation (RR 0.77, 95% CI [0.53–1.12], *p* = 0.17; I^2^ = 38%) (Fig. S8), breath-holding (RR 1.40, 95% CI [0.32–6.18], *p* = 0.66; I^2^ = 0%) (Fig. S12), and cough (RR 0.55, 95% CI [0.25–1.22], *p* = 0.14; I^2^ = 73%) (Fig. S13).

### Subgroup analysis

Further, through subgroup analysis, the influence of factors such as the use of anesthetics, body position, and age on the occurrence of airway complications during laryngeal mask removal at different levels of anesthesia was observed.

**Figure 3:**
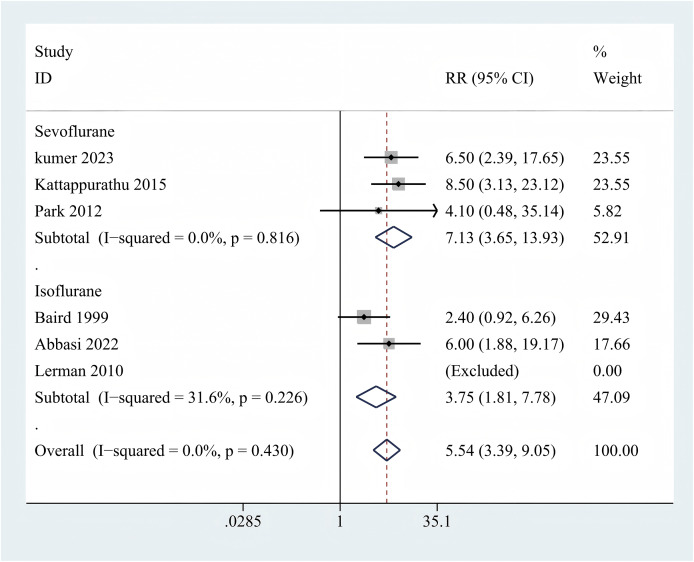
Subgroup analysis by anesthetic agent (sevoflurane/isoflurane) comparing deep *vs*. awake removal for airway obstruction.

Regarding anesthetic agents, no significant differences were observed in overall complications, laryngospasm, desaturation, or cough rates (Figs. S2, S6, S9, S14). However, both agents showed significantly increased airway obstruction risk with deep removal (sevoflurane: RR 7.13, 95% CI [3.65–13.93]; isoflurane: RR 3.75, 95% CI [1.81–7.78]; all *p* < 0.001) (Fig. 3).

**Figure 4:**
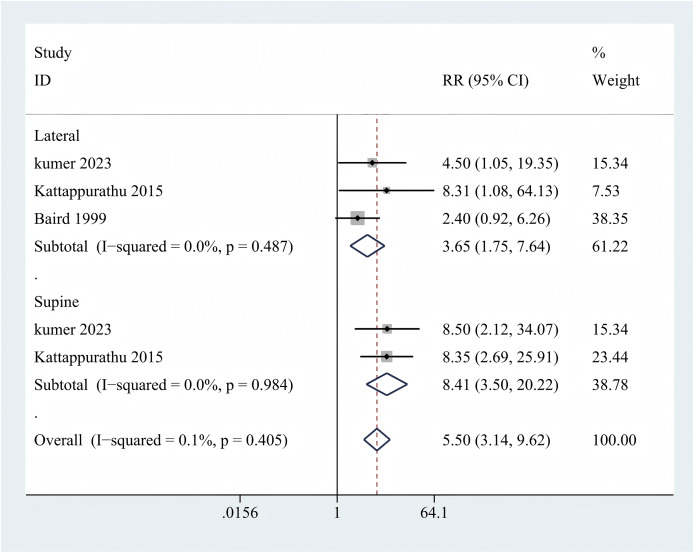
Subgroup analysis by patient positioning (supine position/lateral position) comparing deep *vs*. awake removal for airway obstruction.

For patient positioning, both lateral and supine positions similarly reduced secretions during deep removal (lateral: RR 0.07, 95% CI [0.02–0.25]; supine: RR 0.03, 95% CI [0.01–0.24]; *p* < 0.01) but increased obstruction risk (lateral: RR 3.65, 95% CI [1.75–7.64]; supine: RR 8.51, 95% CI [3.50–20.22]; *p* < 0.001) (Figs. 4, S17). Lateral positioning during deep removal significantly reduced desaturation risk (RR 0.46, 95% CI [0.25–0.86]; *p* = 0.014), while supine positioning did not (RR 1.25, 95% CI [0.66–2.35]) (Fig. S10). No position-dependent differences were noted for overall complications (Fig. S3).

**Figure 5:**
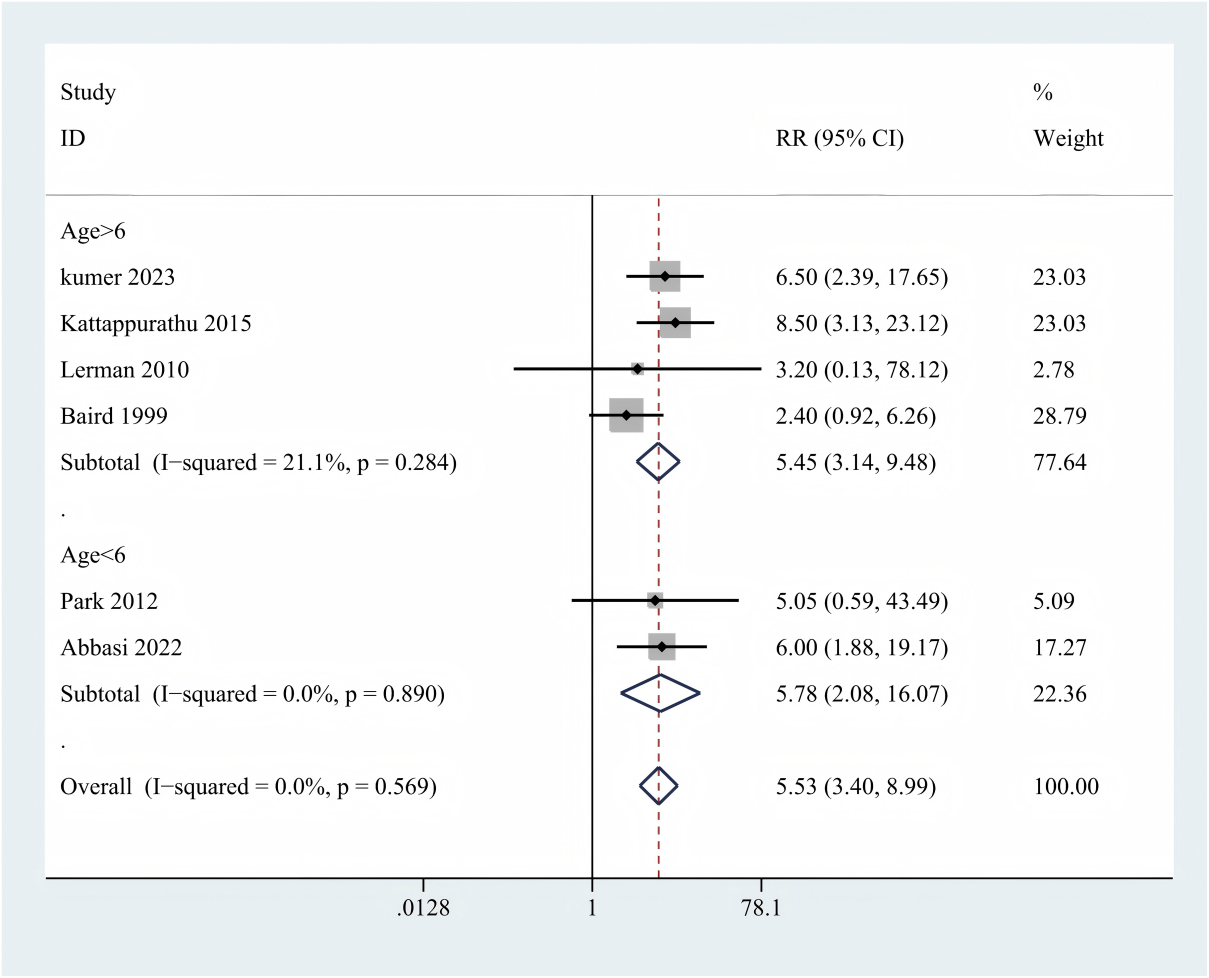
Subgroup analysis by patient age (<6/>6) comparing deep *vs*. awake removal for airway obstruction.

For age groups, no significant differences were found in overall complications, laryngospasm, desaturation, secretions, or cough rates (Figs. S4, S7, S11, S18). Both age groups exhibited significantly increased airway obstruction risk with deep removal (>6 years: RR 5.45, 95% CI [3.14–9.48]; <6 years: RR 5.78, 95% CI [2.08–16.07]; all *p* < 0.001) (Fig. 5).

### Sensitivity analysis and publication bias

Sensitivity analysis for overall complications indicated that no single study significantly altered the pooled relative risk (RR) or its associated 95% confidence intervals (CIs) (Fig. S20). Furthermore, visual inspection of the funnel plot, which displayed the standard error of log RR against log RR, exhibited symmetry (Fig. S21), suggesting no significant publication bias. This absence of statistically significant publication bias was further confirmed by both Egger’s test (*p* = 0.127) and Begg’s test (*p* = 0.128) for overall complications.

## Discussion

This meta-analysis of 10 randomized controlled trials suggests comparable overall airway complication rates between deep and awake LMA removal in pediatric patients. However, this aggregate equivalence conceals a notable trade-off in specific outcomes: deep removal was associated with a higher risk of airway obstruction (RR 5.53), whereas awake removal was linked to a higher incidence of secretion-related complications. No statistically significant differences were observed between the two techniques regarding the risks of laryngospasm or desaturation.

It is important to recognize that the primary outcome of “overall complications” aggregates events of vastly different clinical severity—equating manageable physiological responses like excessive secretions with potentially life-threatening emergencies such as airway obstruction. Our findings suggest that deep removal may be associated with a risk profile characterized by low-frequency but high-severity events. Therefore, the lack of a statistical difference in the composite outcome should not be misconstrued as evidence of equivalent safety. Given that acute airway obstruction poses a more immediate threat to patient safety compared to manageable secretions, prioritizing the prevention of obstruction appears to be a prudent clinical strategy.

In the context of this elevated obstruction risk, patient positioning emerged as a relevant factor. Lateral positioning during deep removal was associated with a 54% lower risk of desaturation while preserving secretion control, whereas supine positioning was linked to a substantially higher obstruction risk (RR 8.51). However, interpretation requires caution because positioning was rarely randomized in the included trials. Consequently, the observed benefits may be confounded by unmeasured variables such as clinician vigilance or institutional practice patterns. Despite this limitation, lateral positioning serves as a prudent risk-mitigation strategy rather than a definitive protective guarantee. Mechanistically, this position helps maintain airway patency by reducing gravity-dependent airway closure and countering anesthetic-induced pharyngeal collapse.

Determining the optimal timing for LMA removal requires integrating considerations of anesthetic depth, airway anatomy, and physiological reserve. Although infants and young children generally exhibit greater airway collapsibility due to immature anatomy (Jordan et al., 2017), our subgroup analysis suggests that age ≥6 years does not serve as a definitive protective factor against airway obstruction during deep removal, as similarly high risks were observed in children both under and over 6 years of age (RR 5.78 *vs*. 5.45). However, this finding warrants cautious interpretation because the confidence intervals for the <6 years subgroup were wide and infants younger than 2 years were underrepresented in the included trials. Therefore, while current evidence implies that age ≥6 years is not a guarantee of safety, it does not exclude the possibility of inherently higher risks in infants or children with syndromic features. Consequently, clinical practice should prioritize the prevention of severe complications: deep removal should be reserved for scenarios where secretion or cough suppression is prioritized (*e.g*., reactive airway disease) and must be accompanied by lateral positioning and continuous monitoring. Awake removal remains the preferred strategy for obstruction-prone patients, such as those with severe obstructive sleep apnea (OSA) or morbid obesity. These conclusions specifically apply to non-airway surgeries (*e.g*., abdominal/limb procedures). Our analysis intentionally restricted this context to isolate LMA removal depth effects from confounders inherent to airway surgeries. In procedures like tonsillectomy (von Ungern-Sternberg et al., 2010; Cozowicz & Memtsoudis, 2021), altered anatomy, increased bleeding/coughing risks (Merna, Lin & Bhattacharyya, 2019; Jordan et al., 2017; Sjölander et al., 2022), and frequent pre-existing obstruction (Jordan et al., 2017) with postoperative sequelae (Sjölander et al., 2022) fundamentally alter complication profiles. The core principle remains: while deep removal does not increase aggregate complications, it shifts risk toward the more severe endpoint of airway obstruction—necessitating prioritization of patient-specific obstruction risks over rigid age thresholds and implementation of compensatory strategies like positioning optimization.

Building upon the 2018 meta-analysis by Koo et al. (2018), which suggested that deep removal may reduce overall airway complications (odds ratio (OR) 0.56, *p* = 0.04) and the risk of coughing/hypoxemia, while potentially increasing the risk of airway obstruction (OR 3.38, *p* = 0.0005), this study extends the findings with three key advances (Clarke, Brice & Chalmers, 2014). First, we quantified a substantially higher obstruction risk increase of 63% (RR 5.53, 95% CI [3.40–8.99], compared to Koo’s OR of 3.38), highlighting its potentially serious nature. Secondly, our subgroup analysis revealed comparable airway obstruction risks between children under 6 years and those 6 years or older (RR 5.78 *vs*. 5.45), challenging the clinical relevance of traditional age thresholds—a critical variable not previously explored. Finally, we identified the lateral position as an innovative compensatory strategy. Employing this position during deep LMA removal appeared to reduce the hypoxemia risk by 54% while preserving the benefit of improved secretion control, thus helping to address a core limitation of deep removal. Collectively, these findings support a shift in clinical practice from solely aiming to “reduce overall complications” towards a stratified risk management approach: prioritizing awake removal for patients prone to obstruction, and reserving deep removal—strictly accompanied by the lateral position and continuous monitoring—for scenarios where secretion control is paramount.

The overall analysis exhibited substantial heterogeneity (I^2^ = 80%), likely stemming from methodological discrepancies in patient populations, anesthesia protocols, and adverse event definitions across the included trials (Lerman et al., 2010; Abbasi, Siddiqui & Qamar-Ul-Hoda, 2022; Kumar et al., 2023; Trachsel et al., 2022; Baird, Mayor & Goodwin, 1999; Chhikara & Seelwal, 2024), particularly regarding the definitions of adverse events and anesthetic protocols. Specifically, thresholds for desaturation varied significantly (*e.g*., SpO_2_ <90% *vs*. <95%). While trials utilizing higher thresholds (<95%) inherently capture a broader spectrum of mild hypoxemic events compared to those using lower thresholds (<90%), this variation primarily influences the absolute event rate rather than the relative treatment effect. Since the definition was applied consistently to both randomized arms within each individual trial, this discrepancy contributes to between-study heterogeneity but is unlikely to introduce systematic bias favoring either technique. Although subgroup analyses partially addressed this heterogeneity, it remains a notable constraint in interpreting the results (von Ungern-Sternberg et al., 2010).

Additionally, most included trials were assessed as having a high or unclear risk of performance bias due to the inherent difficulty of blinding anesthesiologists to the timing of LMA removal. While this lack of blinding may inflate the reporting of subjective outcomes such as coughing or excessive secretions, it is less likely to bias objective, critical endpoints like desaturation (defined by pulse oximetry thresholds) or airway obstruction (requiring physical intervention).

Furthermore, differences in anesthesia maintenance techniques (*e.g*., total intravenous anesthesia *vs*. inhalational anesthesia) may influence airway tone and emergence profiles. However, the majority of included studies predominantly employed volatile agents such as sevoflurane and isoflurane (Lerman et al., 2010; Abbasi, Siddiqui & Qamar-Ul-Hoda, 2022; Sun et al., 2021; Thomas-Kattappurathu, Kasisomayajula & Short, 2015; Baird, Mayor & Goodwin, 1999), with underrepresentation of newer agents like dexmedetomidine and propofol—which are theorized to enhance airway stability and facilitate smoother emergence (Klučka et al., 2015; Karam et al., 2023; Xiao et al., 2022; Wang et al., 2019; Xia et al., 2015). An additional understudied variable is pre-extubation airway management interventions. For instance, applying positive end-expiratory pressure (PEEP) during spontaneous breathing or performing recruitment maneuvers before LMA removal may affect airway obstruction incidence, yet these were inconsistently documented in the reviewed trials. Other factors such as intraoperative fluid management, residual neuromuscular blockade, and opioid administration may confound LMA removal outcomes but were rarely controlled in the studies (Lerman et al., 2010; Abbasi, Siddiqui & Qamar-Ul-Hoda, 2022; Kumar et al., 2023; Thomas-Kattappurathu, Kasisomayajula & Short, 2015; Sinha & Sood, 2006; Baird, Mayor & Goodwin, 1999). These perioperative variables represent opportunities for optimization, independent of anesthetic depth at LMA removal.

## Conclusion

This meta-analysis suggests that light anesthesia removal of the LMA in pediatric patients is associated with a significantly lower risk of airway obstruction compared to deep removal, although it may increase secretion-related complications. Specifically, light anesthesia removal is associated with a 5.5-fold reduction in the risk of airway obstruction, while deep removal reduces secretion-related complications by 96%. Crucially, this significantly increased risk of airway obstruction with deep removal (5.5-fold) was consistently observed across all pediatric age groups, including both children >6 years and those <6 years, challenging the role of rigid age thresholds in clinical decision-making. Lateral positioning emerges as an essential protective strategy, effectively preventing desaturation (reducing risk by 54%) during deep LMA removal without compromising its benefit on secretion control. These findings necessitate individualized protocols that weigh the benefits of reduced secretions (favoring deep removal) against the substantially increased risks of obstruction (favoring light anesthesia removal), prioritizing patient-specific factors over age-based paradigms. Lateral positioning and continuous monitoring should be mandatory when deep removal is employed. Future research must address evidence gaps in novel anesthetic agents, high-risk populations, and standardized depth assessment to optimize pediatric airway safety.

## Supplemental Information

10.7717/peerj.21551/supp-1 Supplemental Information 1 PRISMA checklist.

10.7717/peerj.21551/supp-2 Supplemental Information 2 Raw data.

10.7717/peerj.21551/supp-3 Supplemental Information 3 Forest plot of comparison between deep removal (experimental) and awake removal (control) for the outcome of overall complications.

10.7717/peerj.21551/supp-4 Supplemental Information 4 Forest plot of comparison between deep removal (experimental) and awake removal (control) for the outcome of airway obstruction.

10.7717/peerj.21551/supp-5 Supplemental Information 5 Subgroup Analysis by patient positioning (supine position/lateral position) comparing Deep *vs*. Awake Removal for overall complications.

10.7717/peerj.21551/supp-6 Supplemental Information 6 Subgroup Analysis by patient age (<6/>6) comparing Deep *vs*. Awake Removal for overall complications.

10.7717/peerj.21551/supp-7 Supplemental Information 7 Forest plot of comparison between deep removal (experimental) and awake removal (control) for the outcome of laryngospasm.

10.7717/peerj.21551/supp-8 Supplemental Information 8 Subgroup Analysis by Anesthetic Agent (sevoflurane/isoflurane) comparing Deep *vs*. Awake Removal for laryngospasm.

10.7717/peerj.21551/supp-9 Supplemental Information 9 Subgroup Analysis by patient age (<6/>6) comparing Deep *vs*. Awake Removal for laryngospasm.

10.7717/peerj.21551/supp-10 Supplemental Information 10 Forest plot of comparison between deep removal (experimental) and awake removal (control) for the outcome of desaturation.

10.7717/peerj.21551/supp-11 Supplemental Information 11 Subgroup Analysis by Anesthetic Agent (sevoflurane/isoflurane) comparing Deep *vs*. Awake Removal for desaturation.

10.7717/peerj.21551/supp-12 Supplemental Information 12 Subgroup Analysis by patient positioning (supine position/lateral position) comparing Deep *vs*. Awake Removal for desaturation.

10.7717/peerj.21551/supp-13 Supplemental Information 13 Subgroup Analysis by patient age (<6/>6) comparing Deep *vs*. Awake Removal for desaturation.

10.7717/peerj.21551/supp-14 Supplemental Information 14 Forest plot of comparison between deep removal (experimental) and awake removal (control) for the outcome of breath-holding.

10.7717/peerj.21551/supp-15 Supplemental Information 15 Forest plot of comparison between deep removal (experimental) and awake removal (control) for the outcome of cough.

10.7717/peerj.21551/supp-16 Supplemental Information 16 Subgroup Analysis by Anesthetic Agent (sevoflurane/isoflurane) comparing Deep *vs*. Awake Removal for cough.

10.7717/peerj.21551/supp-17 Supplemental Information 17 Subgroup Analysis by patient age (<6/>6) comparing Deep *vs*. Awake Removal for cough.

10.7717/peerj.21551/supp-18 Supplemental Information 18 Forest plot of comparison between deep removal (experimental) and awake removal (control) for the outcome of excessive secretions.

10.7717/peerj.21551/supp-19 Supplemental Information 19 Subgroup Analysis by patient positioning (supine position/lateral position) comparing Deep *vs*. Awake Removal for secretions.

10.7717/peerj.21551/supp-20 Supplemental Information 20 Subgroup Analysis by patient age (<6/>6) comparing Deep *vs*. Awake Removal for secretions.

10.7717/peerj.21551/supp-21 Supplemental Information 21 Risk of bias graph.

10.7717/peerj.21551/supp-22 Supplemental Information 22 Sensitivity analysis for overall complications.

10.7717/peerj.21551/supp-23 Supplemental Information 23 Funnel plot of publication bias for overall complications.

10.7717/peerj.21551/supp-24 Supplemental Information 24 Intended audience.

## Additional Information and Declarations

### Data Availability

The following information was supplied regarding data availability:

This is a Systematic review/Meta-analysis.
