# Supplementary figures and images for: Effects of laryngeal mask airway removal under different anesthesia states on pediatric airway complications: a systematic review and meta-analysis
Source: PeerJ. 2026 Jul 31;14:e21551. doi: 10.7717/peerj.21551 (PMC13431292; doi:10.7717/peerj.21551)

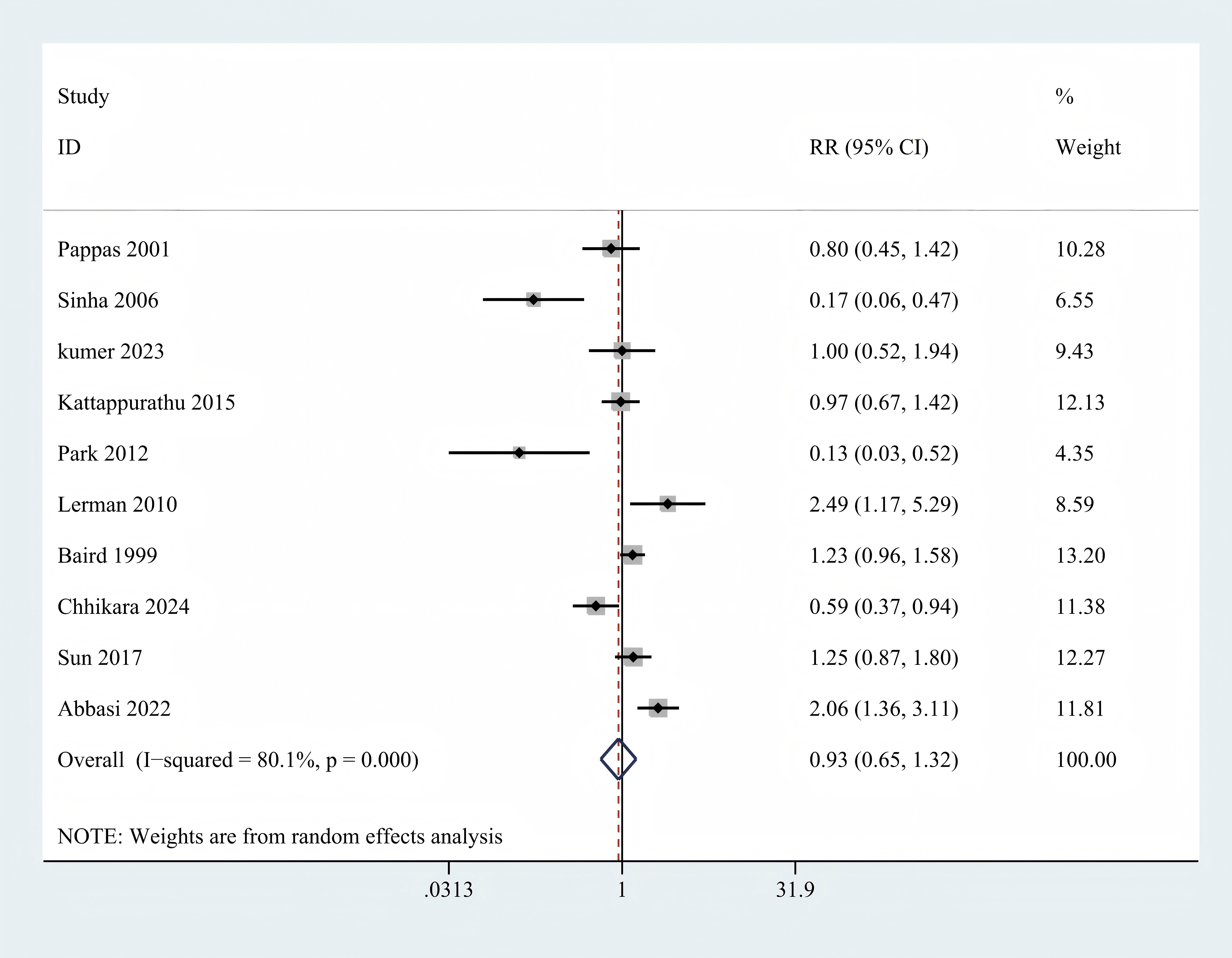

Supplement: Supplemental Information 3 [file peerj-14-21551-s003.png]

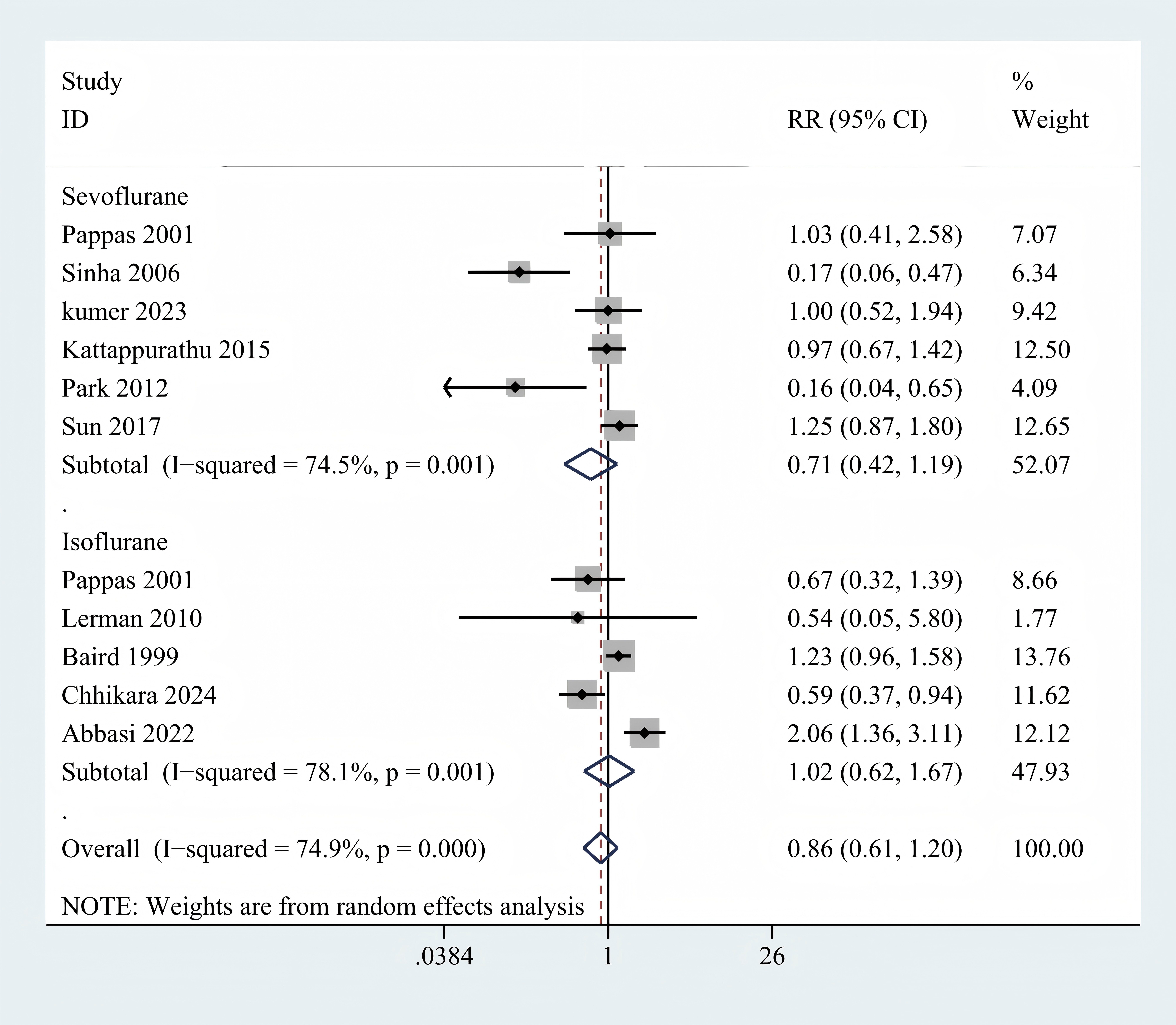

Supplement: Supplemental Information 4 [file peerj-14-21551-s004.png]

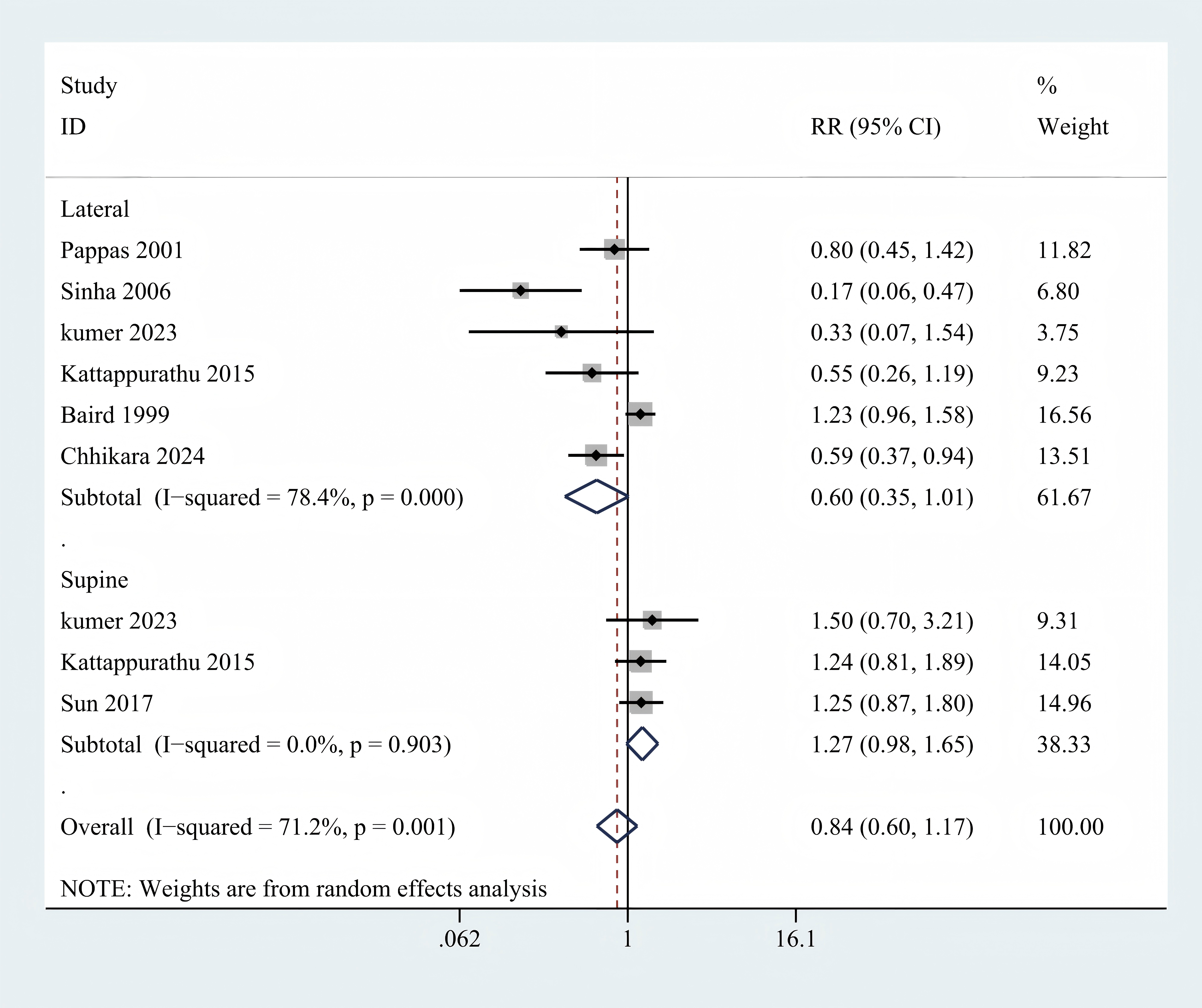

Supplement: Supplemental Information 5 [file peerj-14-21551-s005.png]

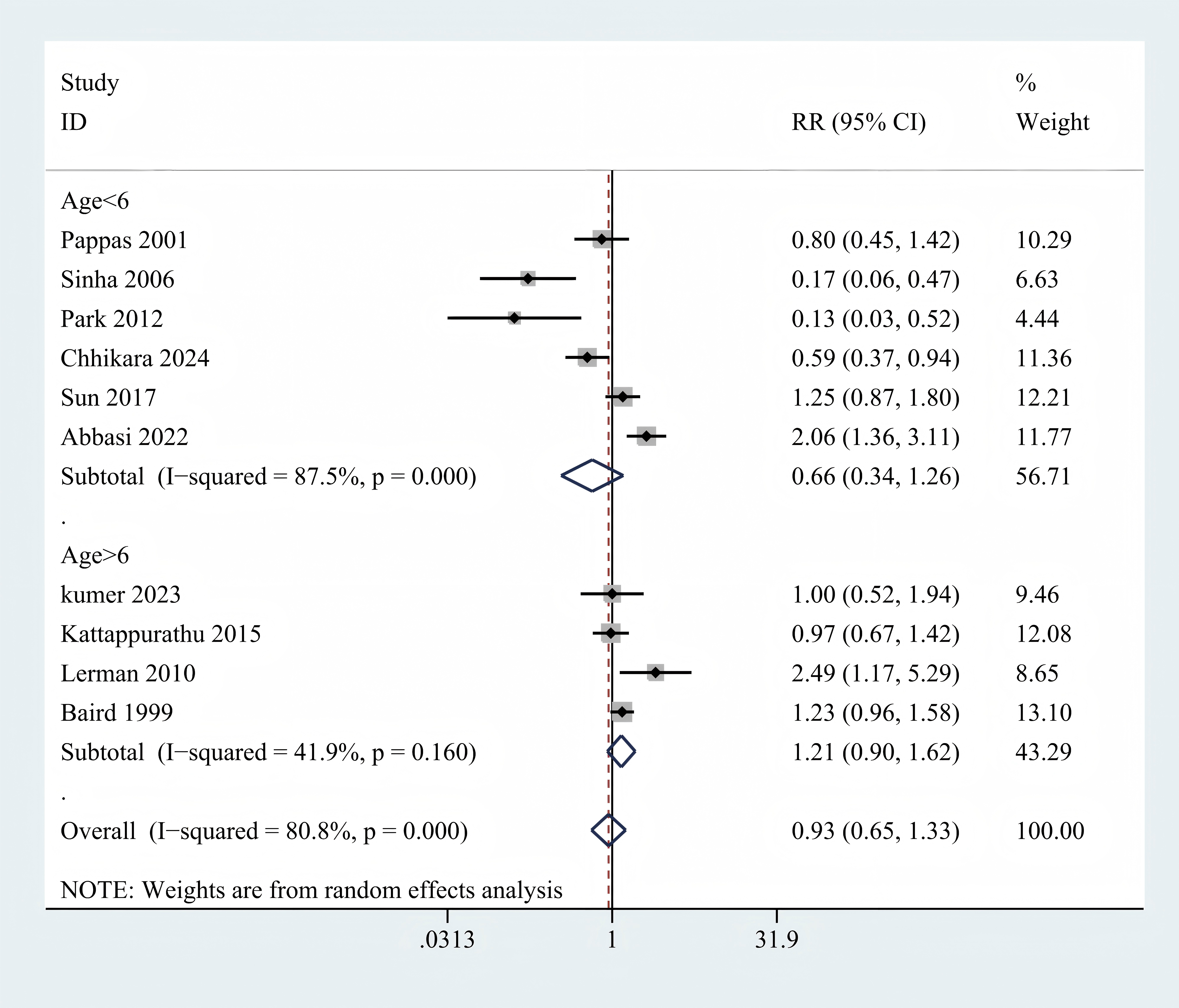

Supplement: Supplemental Information 6 [file peerj-14-21551-s006.png]

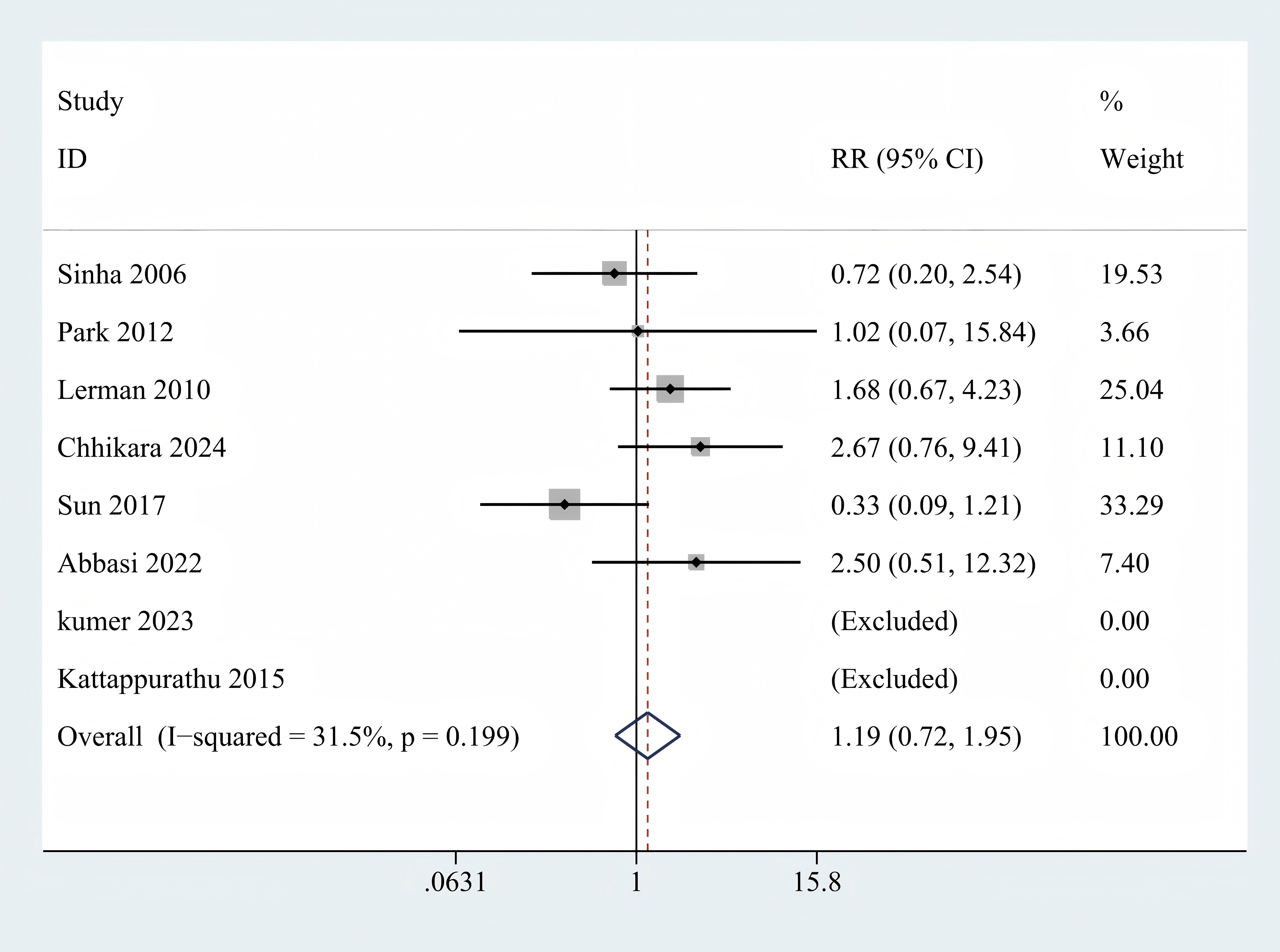

Supplement: Supplemental Information 7 [file peerj-14-21551-s007.png]

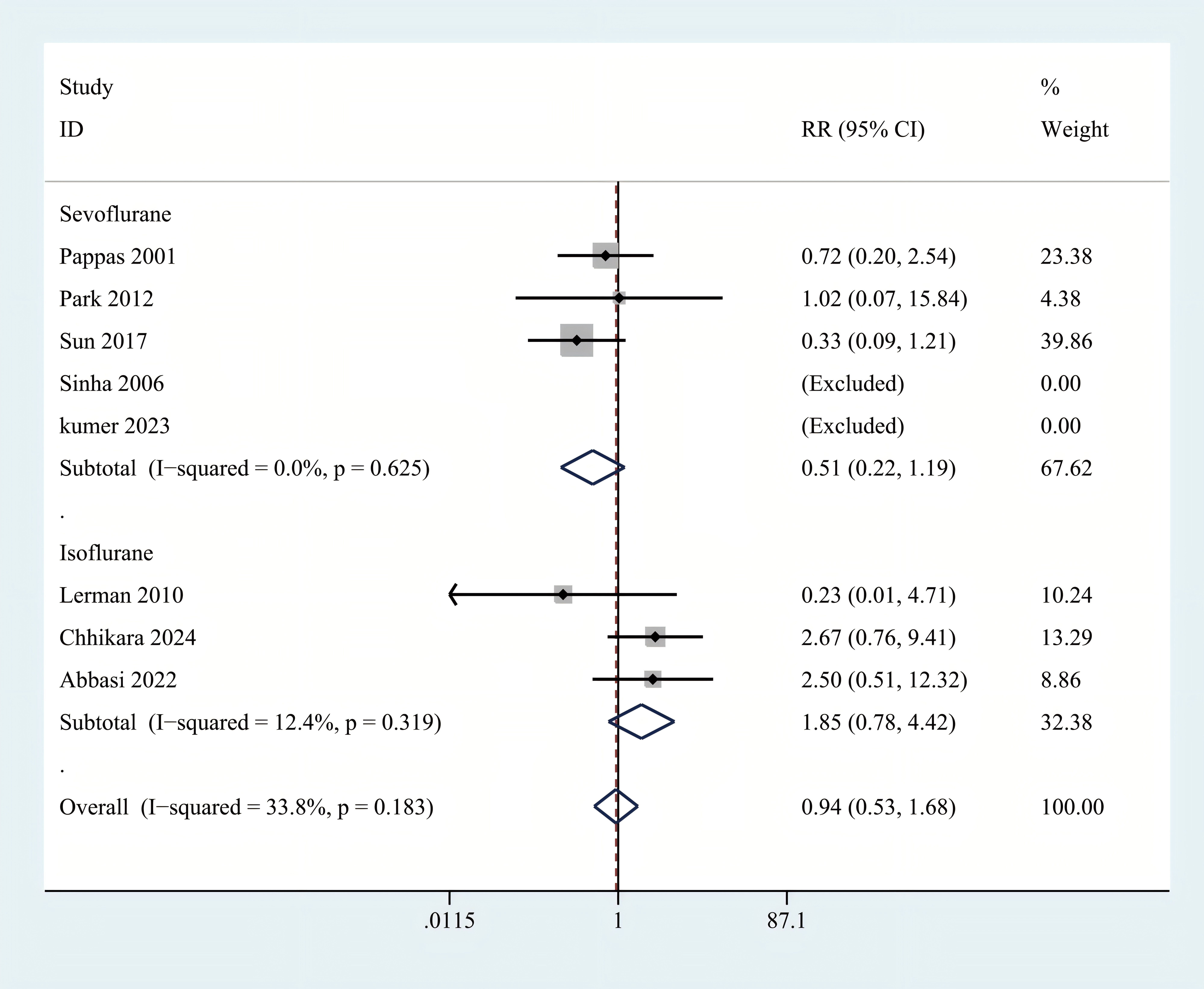

Supplement: Supplemental Information 8 [file peerj-14-21551-s008.png]

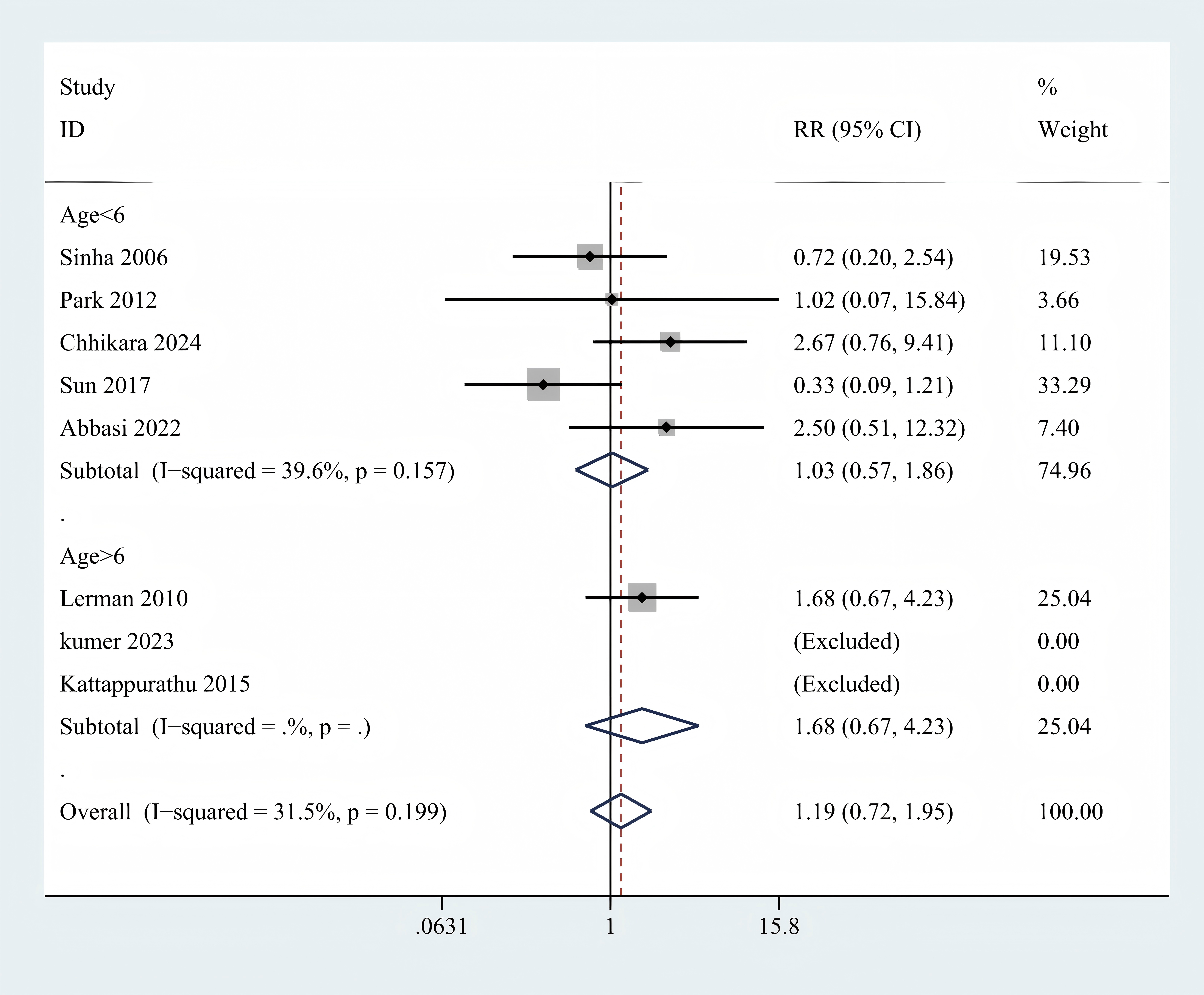

Supplement: Supplemental Information 9 [file peerj-14-21551-s009.png]

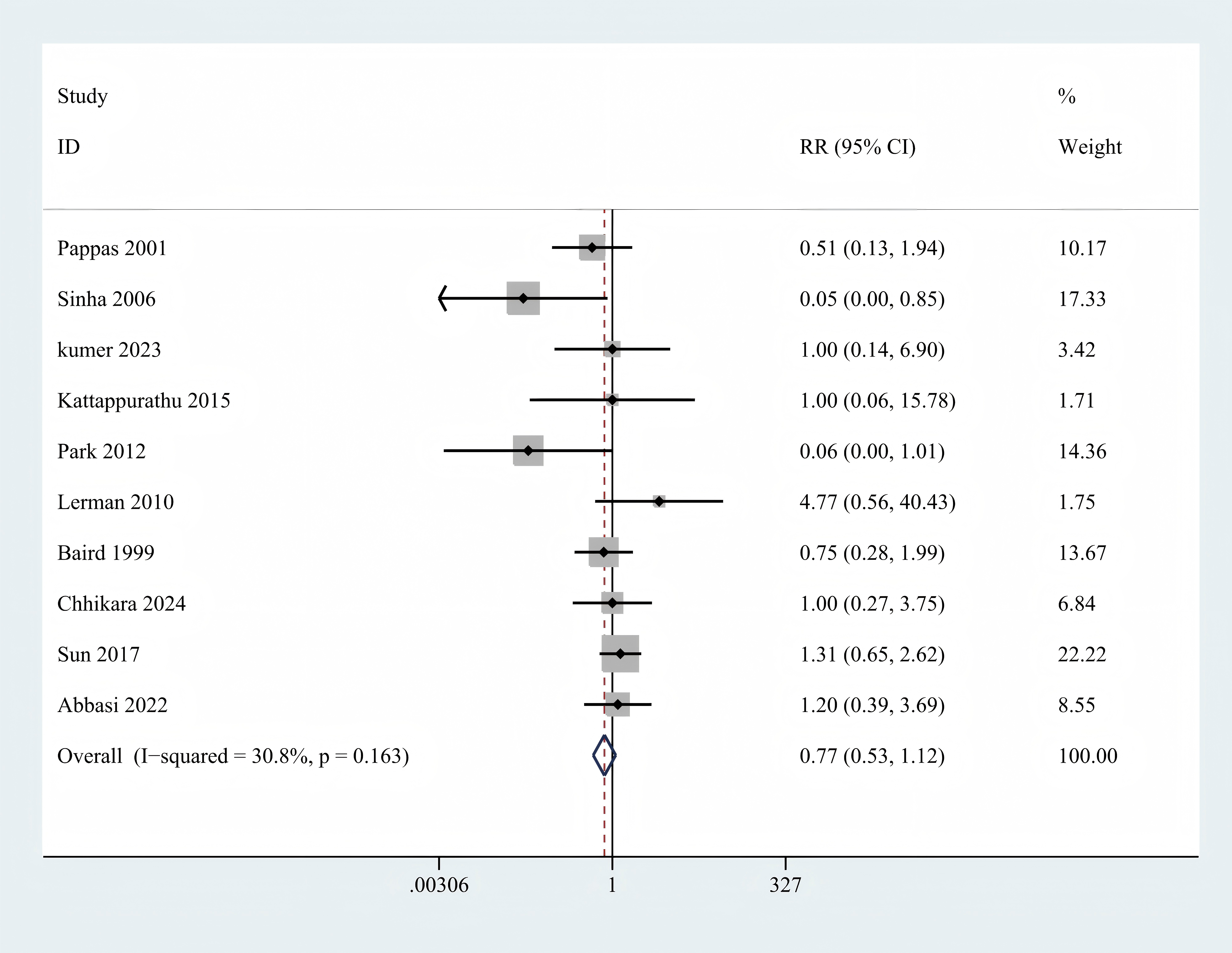

Supplement: Supplemental Information 10 [file peerj-14-21551-s010.png]

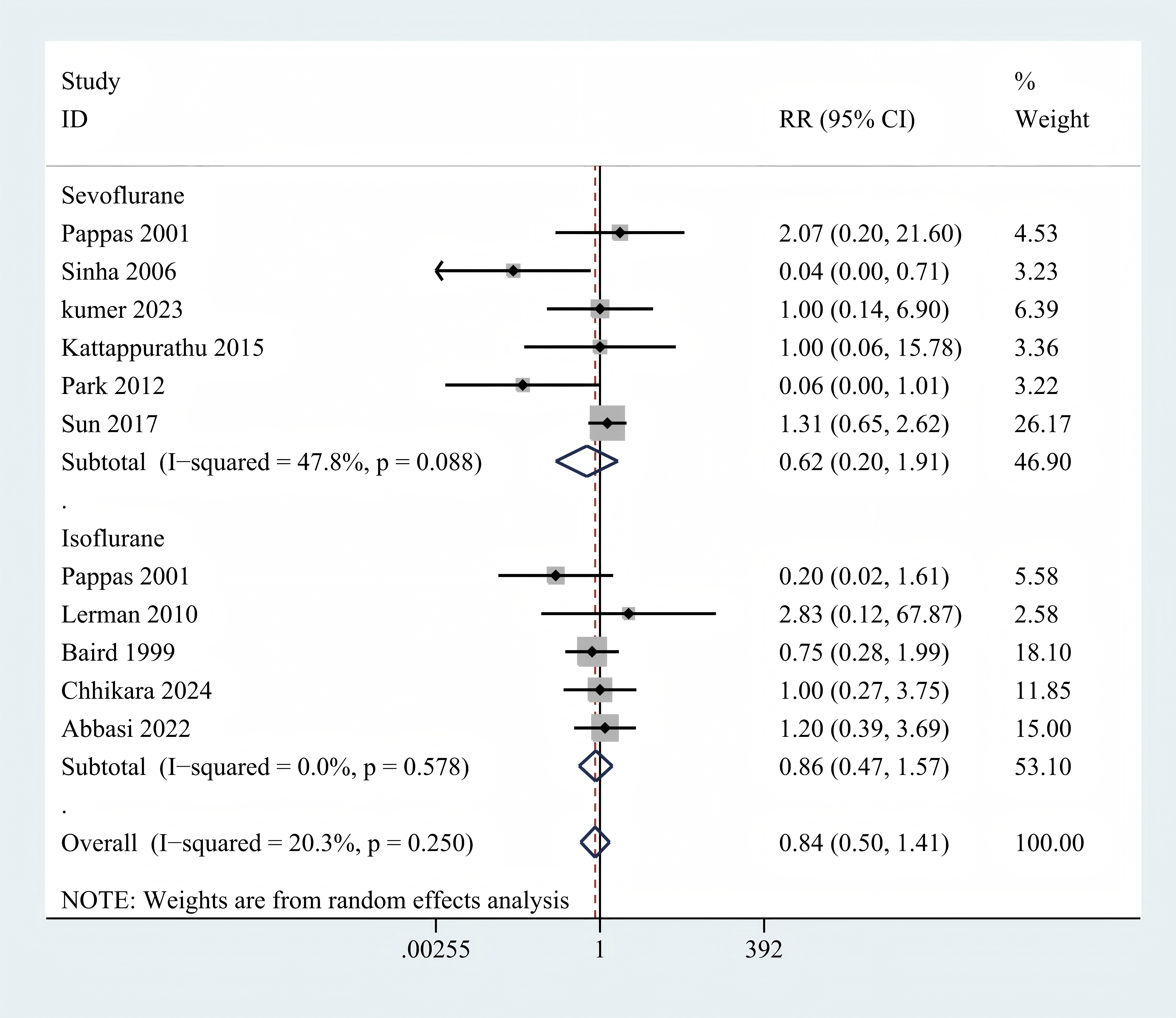

Supplement: Supplemental Information 11 [file peerj-14-21551-s011.png]

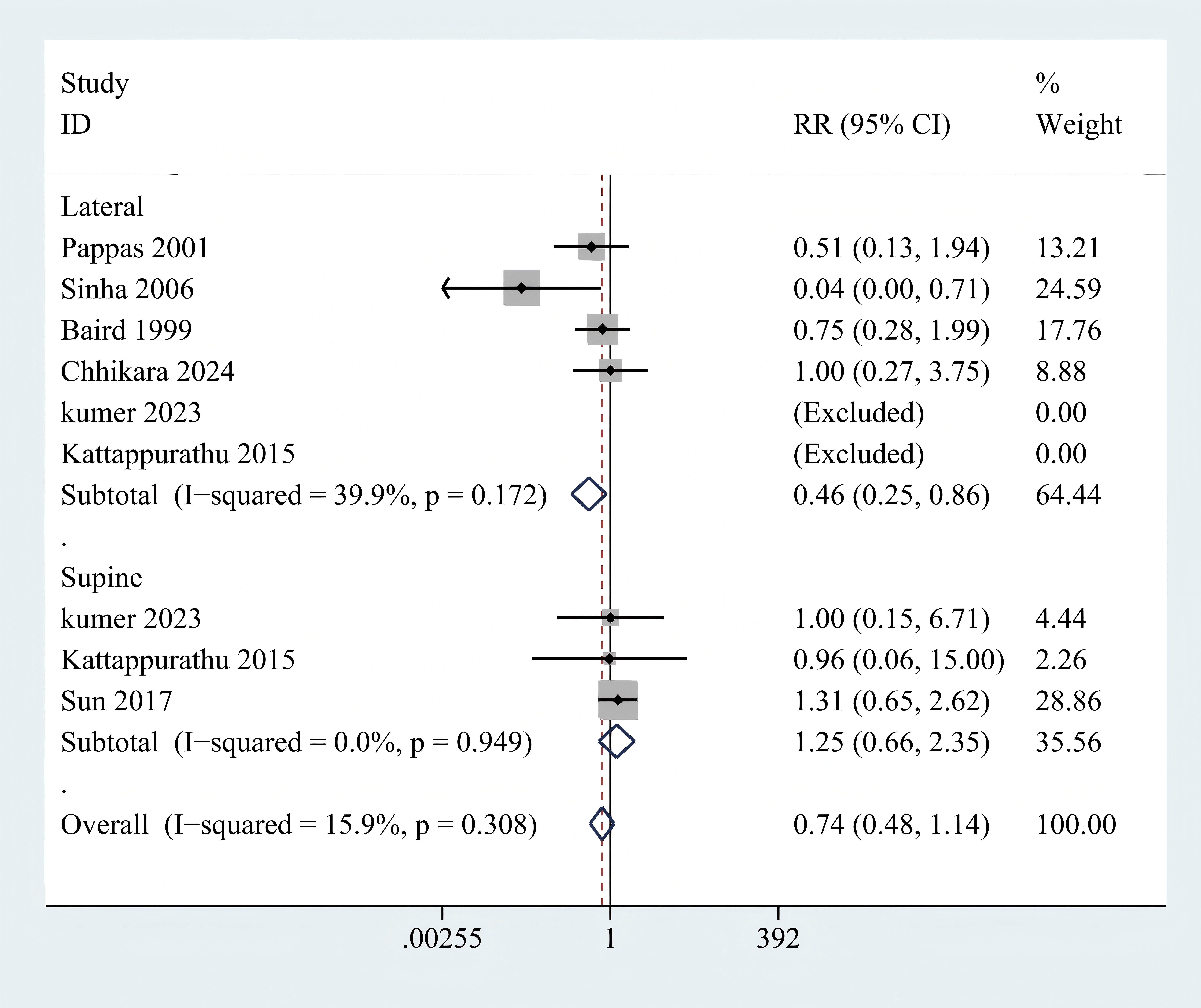

Supplement: Supplemental Information 12 [file peerj-14-21551-s012.png]

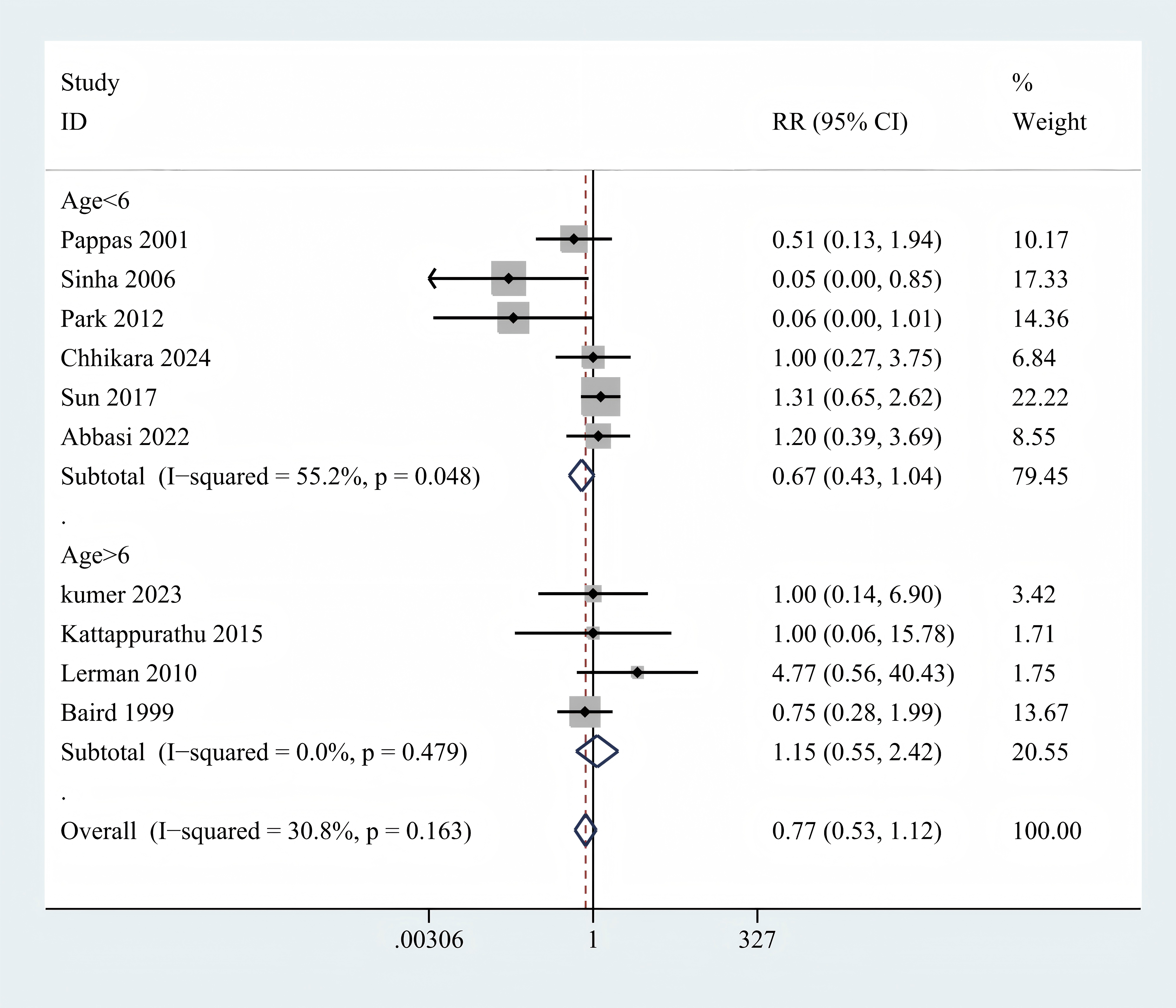

Supplement: Supplemental Information 13 [file peerj-14-21551-s013.png]

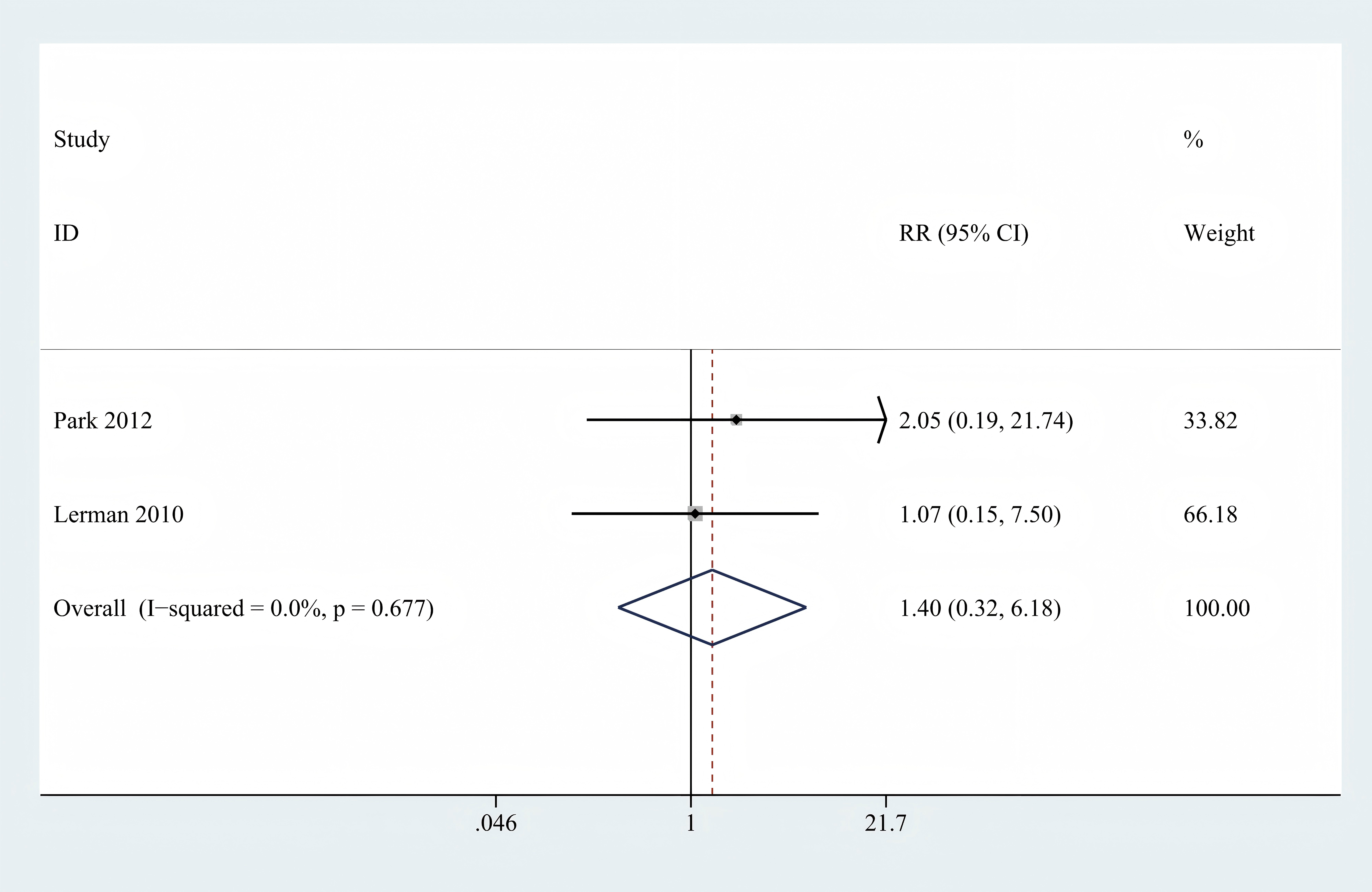

Supplement: Supplemental Information 14 [file peerj-14-21551-s014.png]

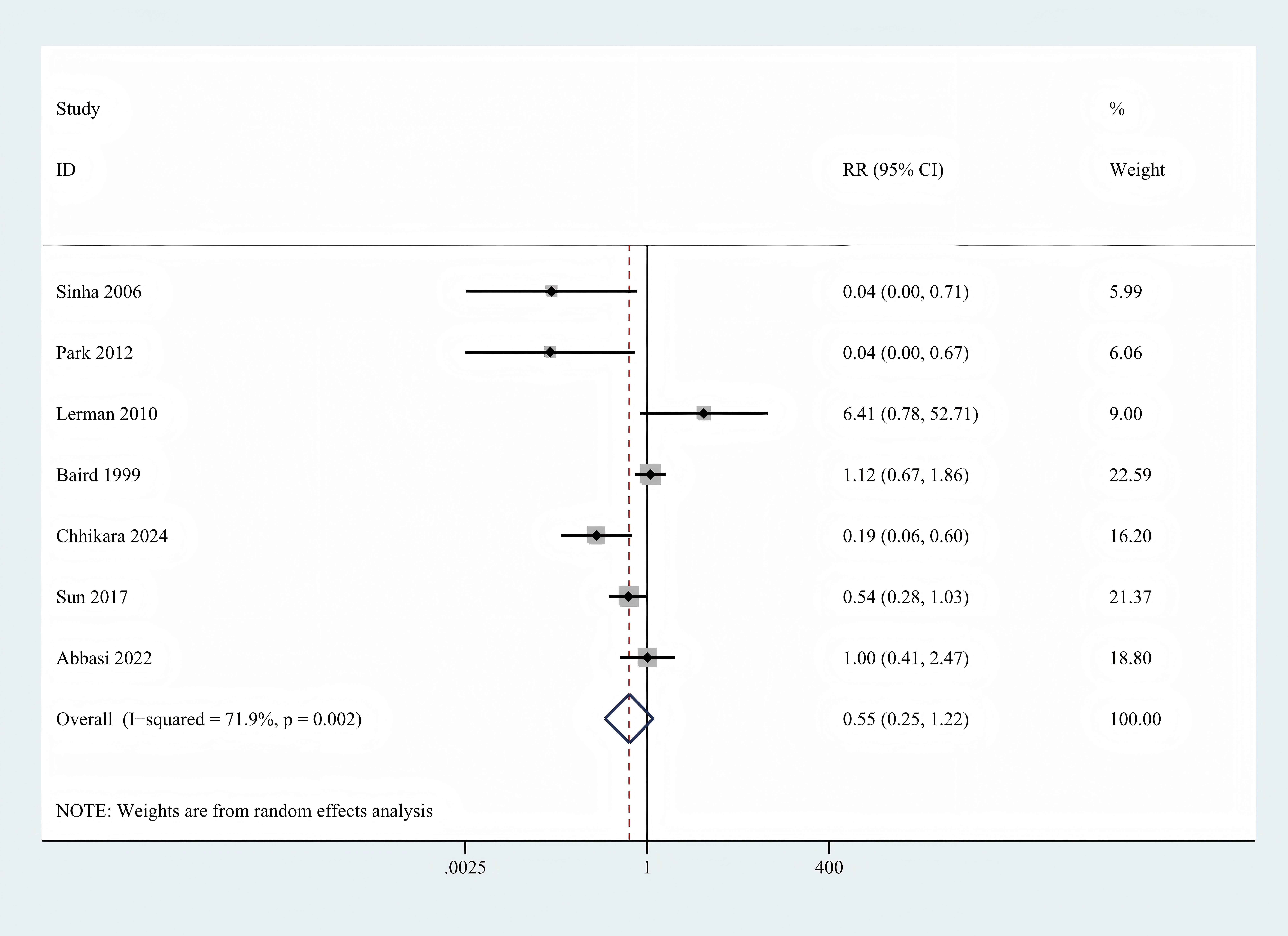

Supplement: Supplemental Information 15 [file peerj-14-21551-s015.png]

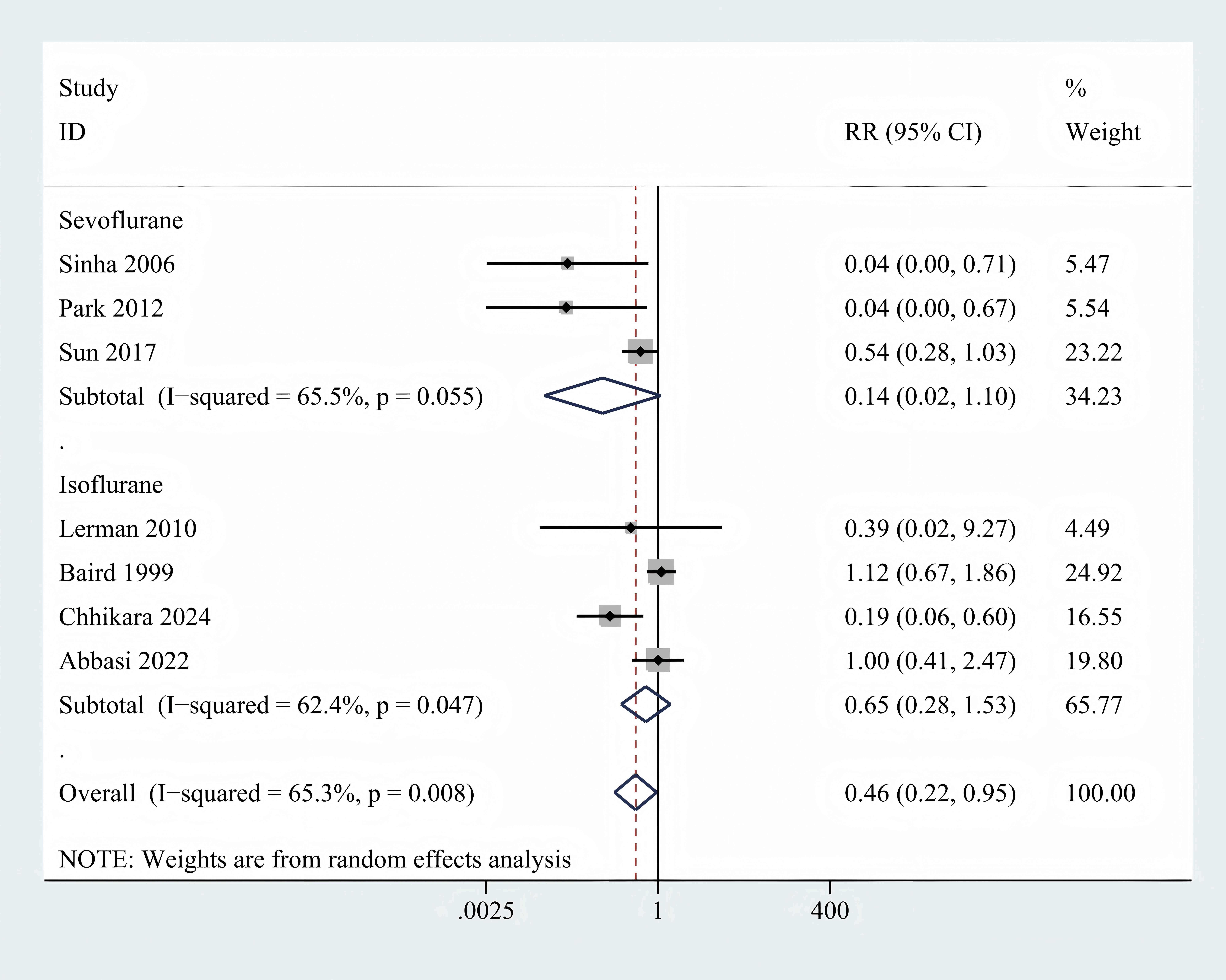

Supplement: Supplemental Information 16 [file peerj-14-21551-s016.png]

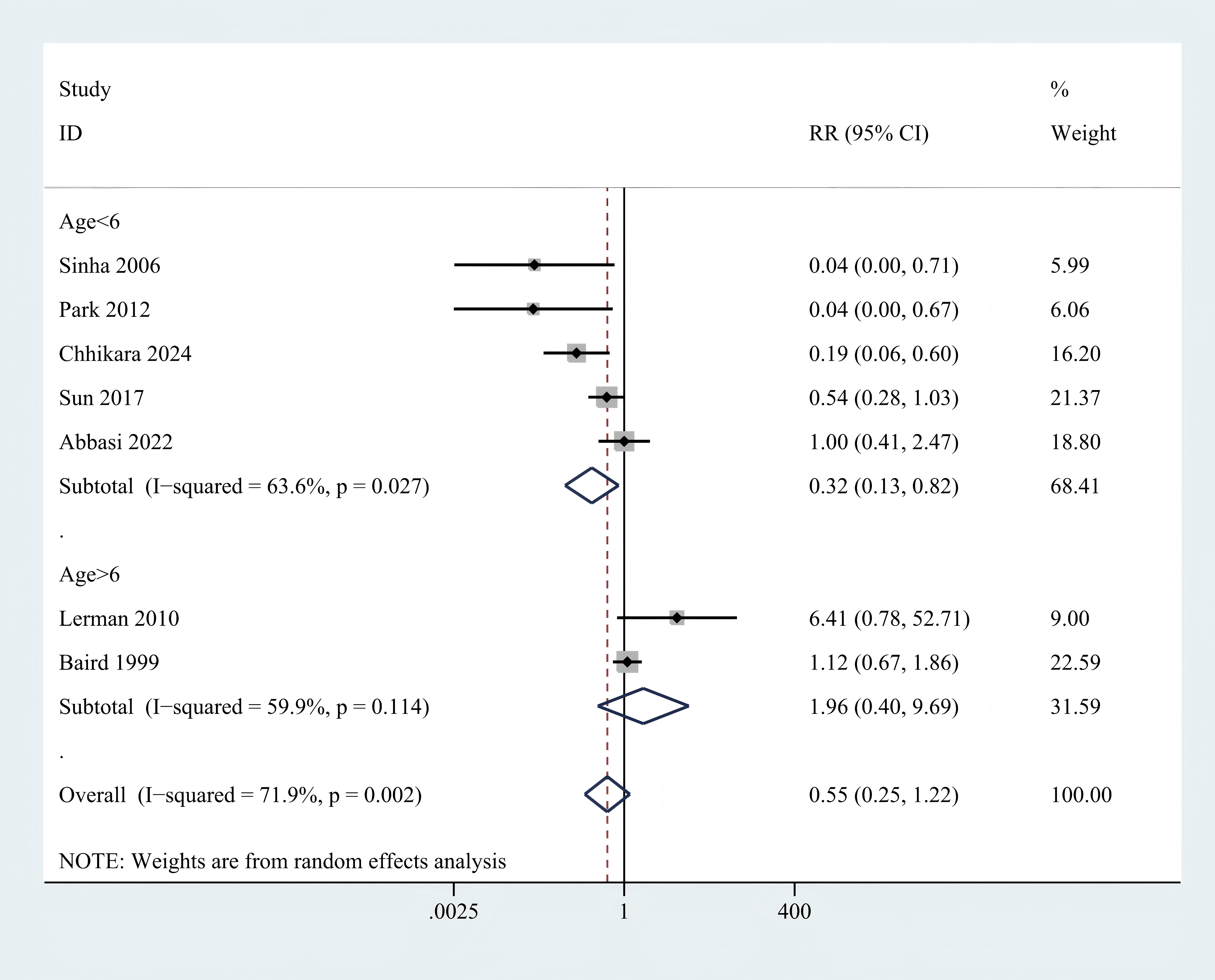

Supplement: Supplemental Information 17 [file peerj-14-21551-s017.png]

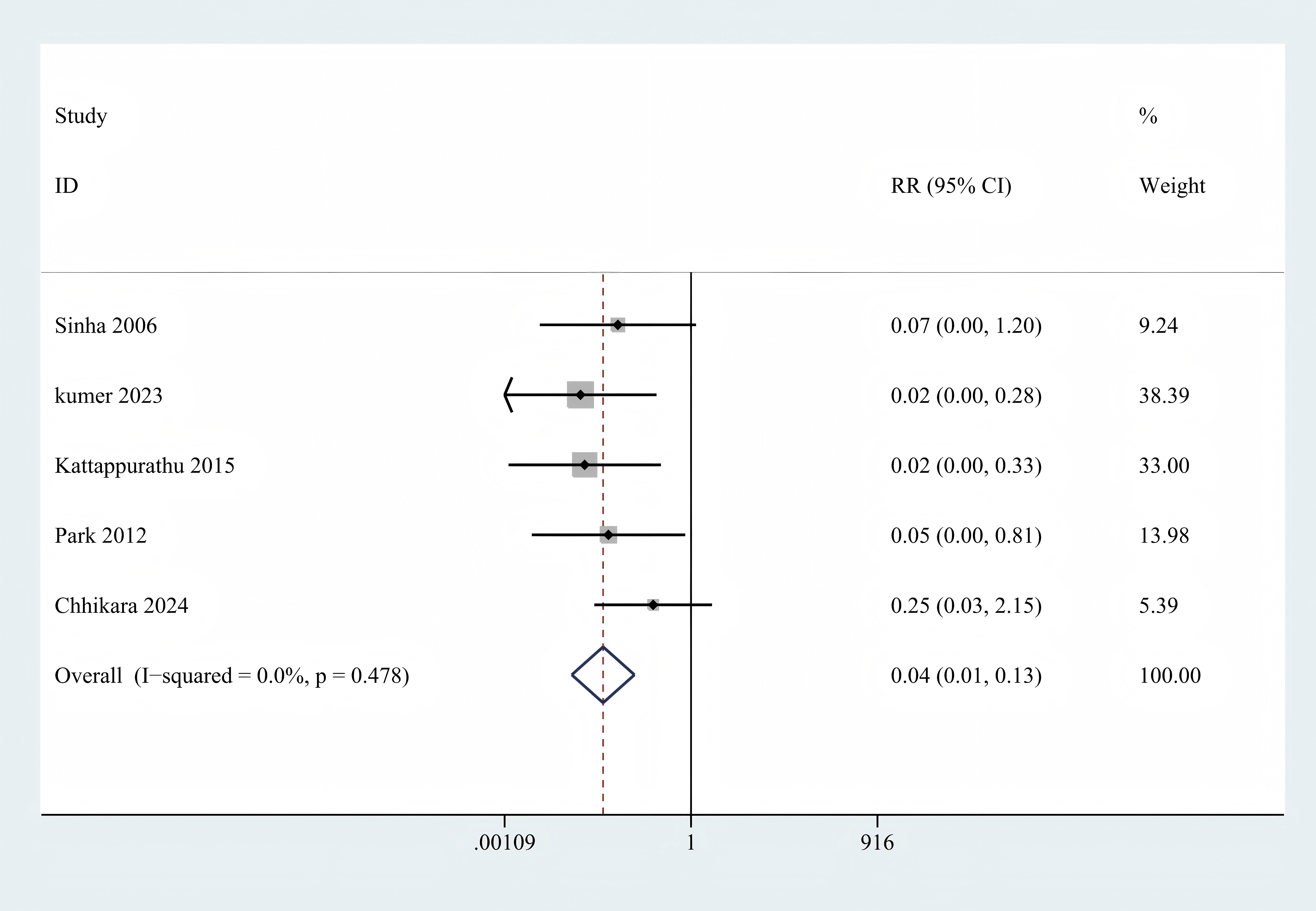

Supplement: Supplemental Information 18 [file peerj-14-21551-s018.png]

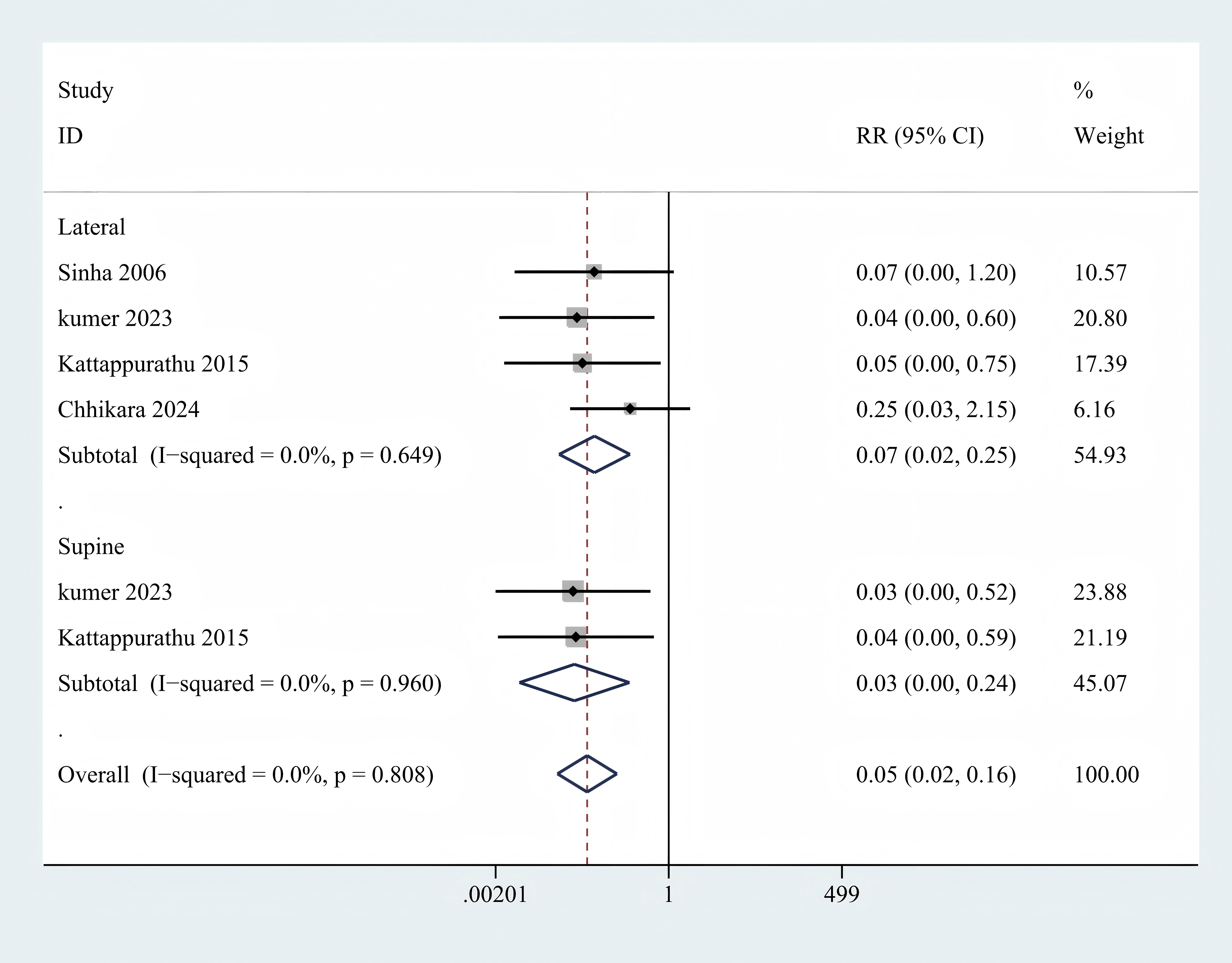

Supplement: Supplemental Information 19 [file peerj-14-21551-s019.png]

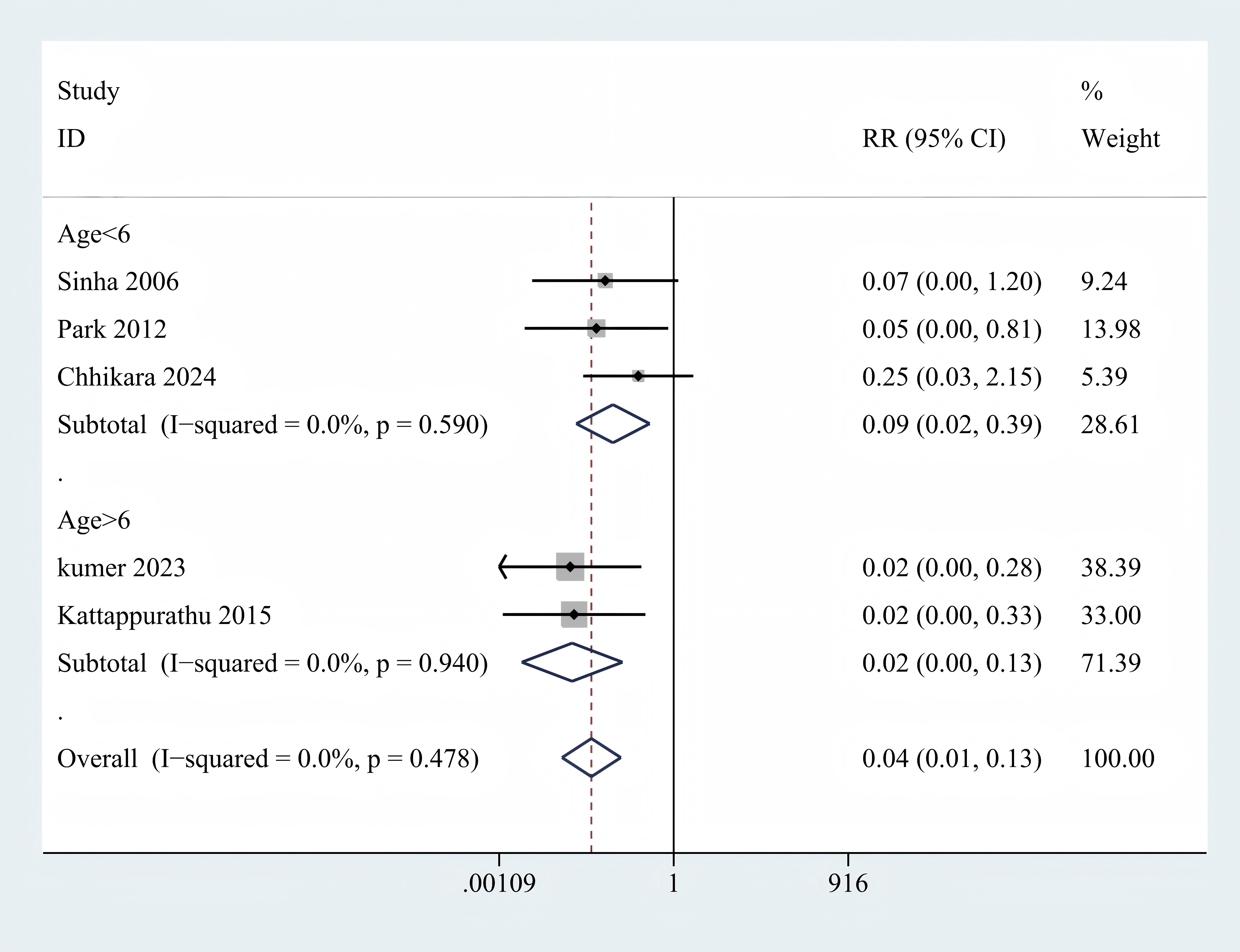

Supplement: Supplemental Information 20 [file peerj-14-21551-s020.png]

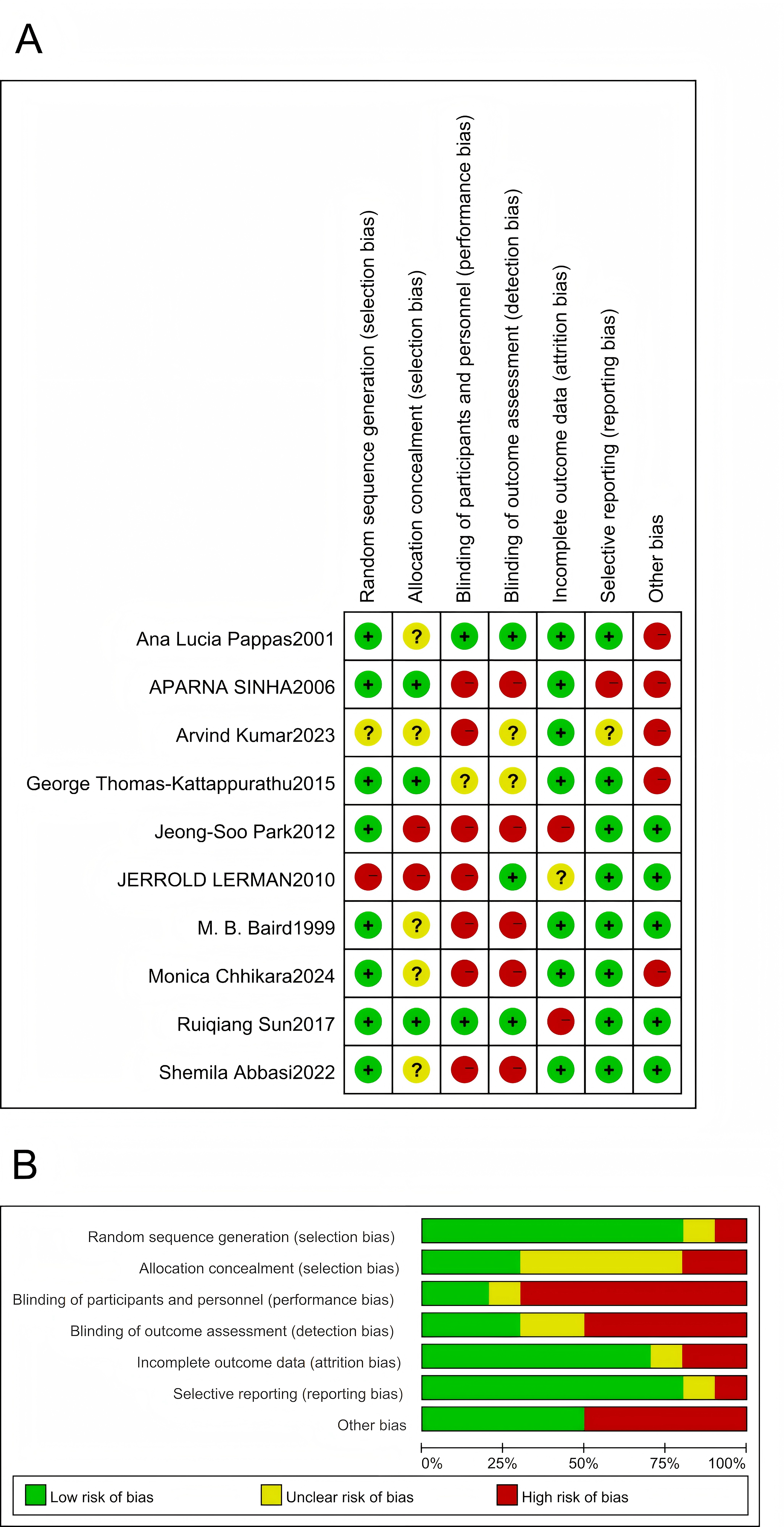

Supplement: Supplemental Information 21 [file peerj-14-21551-s021.png]

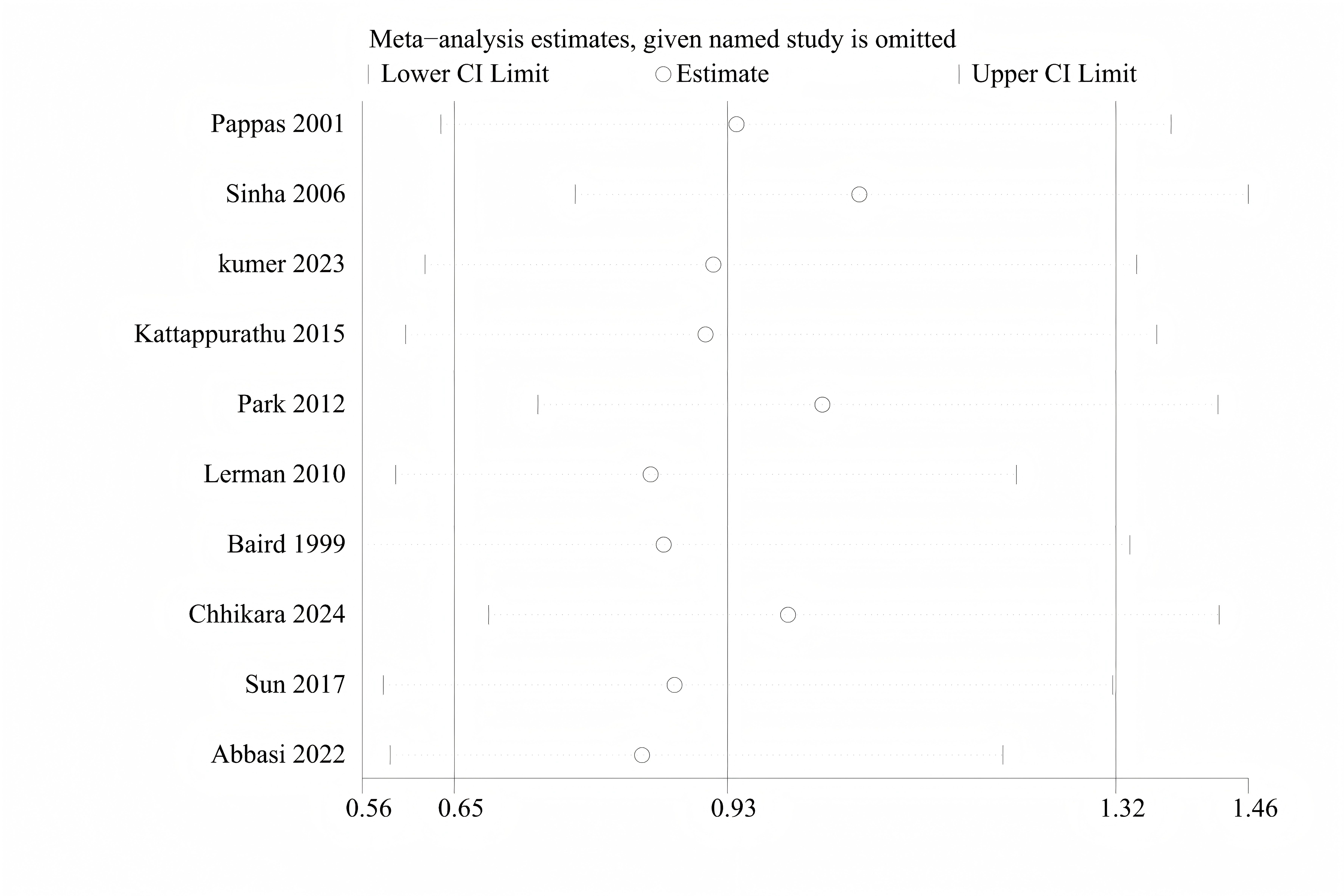

Supplement: Supplemental Information 22 [file peerj-14-21551-s022.png]

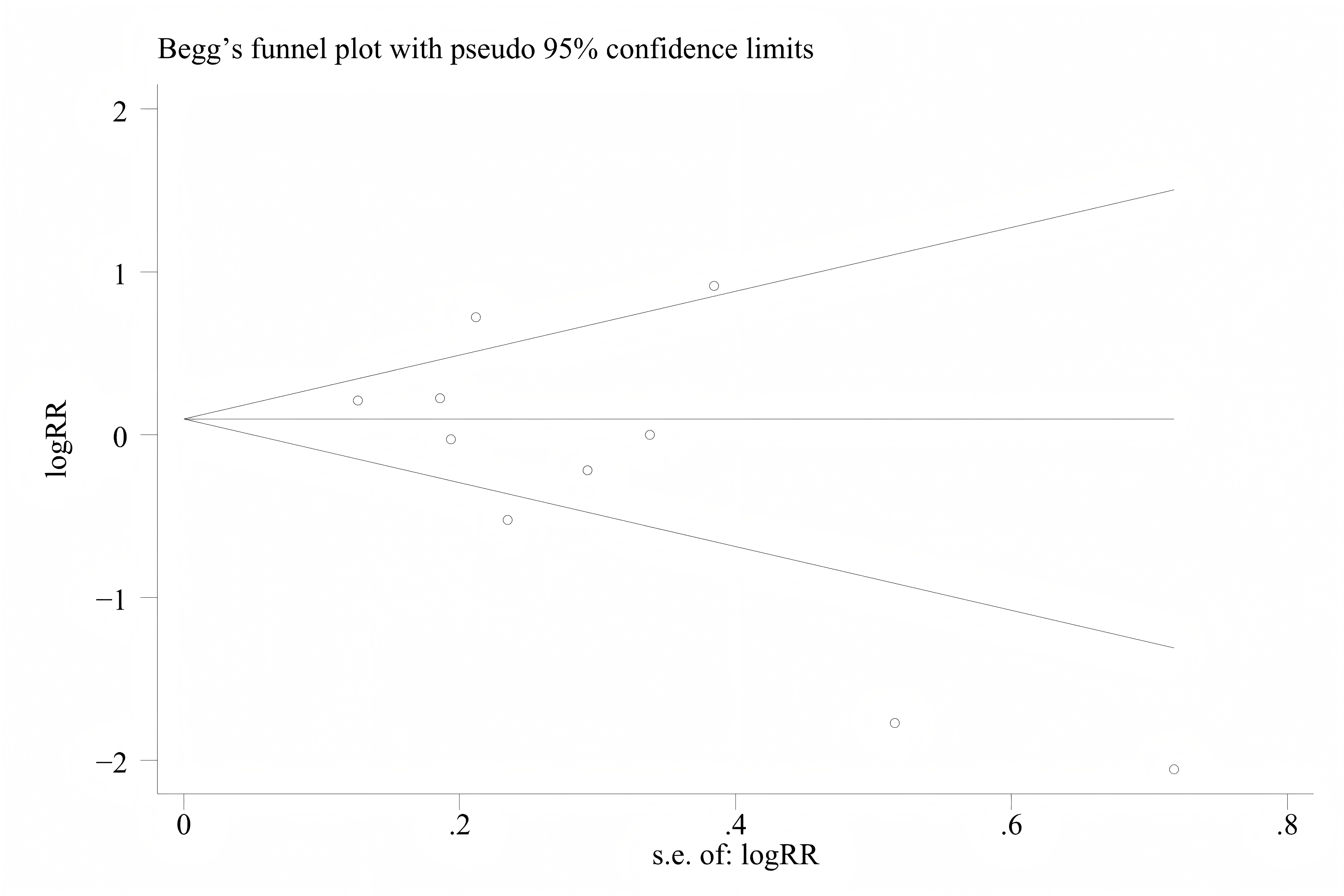

Supplement: Supplemental Information 23 [file peerj-14-21551-s023.png]
