# Supplementary material for: Effects of laryngeal mask airway removal under different anesthesia states on pediatric airway complications: a systematic review and meta-analysis
Source: PeerJ. 2026 Jul 31;14:e21551. doi: 10.7717/peerj.21551 (PMC13431292; doi:10.7717/peerj.21551)
Supplement: Supplemental Information 24 [file peerj-14-21551-s024.docx]

As an author on this systematic review examining optimal LMA removal timing, I am writing to state explicitly the intended readership.

This review is written for four principal audiences:

1. Practising anaesthetists and anaesthesia providers (consultants, trainees and nurse anaesthetists) who manage supraglottic airways in children on a daily basis and who need an evidence-based, procedure-specific recommendation for when to remove the LMA (awake vs. anaesthetised) to minimise coughing, laryngospasm, desaturation.

2. Anaesthesia department heads, clinical leads and quality-improvement committees who are responsible for writing local SOPs, care pathways and day-surgery discharge criteria and who require a high-grade summary of the literature to decide whether a “deep-removal” or “awake-removal” protocol should be adopted for different surgical populations (e.g., ambulatory vs. in-patient, laparoscopic vs. superficial surgery).

3. Guidelines panels (e.g., Difficult Airway Society, ASA, ESA, APSF, NICE) that are updating national or international recommendations on supraglottic airway management and need a rigorously conducted systematic review and meta-analysis with GRADE assessment to support future guidance statements.

4. Clinical researchers in airway management, peri-operative medicine and patient safety who are designing randomised trials or registry studies on airway complications and who require an up-to-date evidence synthesis, identification of knowledge gaps and a set of patient-important outcomes that remain under-studied.

The review therefore targets both bedside clinicians who make the immediate decision of when to remove the LMA and policy-makers who translate that evidence into institutional or national standards.
